# Establishment and Characterization of an Immortalized Porcine Satellite Cell Line from China Junmu No.1 Pigs

**DOI:** 10.3390/vetsci13060556

**Published:** 2026-06-04

**Authors:** Jing Li, Yu He, Xiaoran Zhang, Jiayi Ning, Dali Wang, Chunyan Bai, Boxing Sun, Shaoxuan Zhang, Shuang Liang, Hao Sun

**Affiliations:** Department of Animal Sciences, College of Animal Sciences, Jilin University, Changchun 130062, China

**Keywords:** immortalized porcine satellite cells, Junmu No.1 pigs, SV40 large T antigen, myogenic differentiation, transcriptomic analysis, muscle development

## Abstract

Junmu No.1 is a commercially important Chinese pig breed, yet stable in vitro models for investigating its muscle development mechanisms and genetic regulation remain lacking; this study aimed to establish an immortalized porcine satellite cell line from Junmu No.1 pigs to address this gap. Primary porcine satellite cells (PSCs) were isolated from a 2-day-old Junmu No.1 piglet and immortalized via lentiviral transduction using the pHAGE-EF1α-eGFP-SV40LT-BleoR vector. The resulting cell line (imPSC-JM) was characterized for morphology, satellite cell marker expression, karyotype stability, myogenic differentiation capacity, and long-term proliferative potential, and RNA sequencing combined with Gene Set Enrichment Analysis (GSEA) was performed to assess transcriptomic fidelity relative to primary PSCs. The imPSC-JM line retained characteristic spindle-shaped satellite cell morphology, consistently expressed PAX7, maintained a normal diploid karyotype (2*n* = 38, XY), and showed stable SV40 large T antigen expression, enabling sustained proliferation exceeding 100 cumulative population doublings while preserving myogenic differentiation and the formation of multinucleated myotubes expressing Desmin, MYHC, and DMD. Transcriptomic profiles were highly concordant with primary PSCs (Pearson r ≥ 0.95; R^2^ = 0.9188; 83.8% of expressed genes unchanged), with key satellite-cell and myogenic regulator genes (PAX7, MYOD1, MYF5, MYOG, MYF6) unaltered, while GSEA revealed upregulation of autophagy and inflammatory signaling and downregulation of ribosome biogenesis. The imPSC-JM line thus provides a reliable experimental platform with high transcriptomic fidelity for studying muscle development and genetic regulation in Junmu No.1 pigs.

## Simple Summary

Skeletal muscle satellite cells are adult stem cells essential for muscle growth, repair, and meat production in livestock. However, primary satellite cells are difficult to maintain in laboratory culture because they stop dividing and spontaneously differentiate after only a few passages, limiting their use in research. The Junmu No.1 White pig is a Chinese commercial breed valued for its rapid growth, high lean meat yield, and strong adaptability, but no stable cell model is currently available for studying muscle development in this breed. In this study, we successfully established a long-term cultivable satellite cell line from Junmu No.1 piglets by introducing the SV40 large T antigen gene through lentiviral delivery. The resulting cell line maintained typical satellite cell features, normal chromosome numbers, and the ability to form muscle fibers in culture, while continuing to proliferate for more than 100 cell divisions. Importantly, its overall gene expression profile remained highly similar to that of primary cells. This new cell line provides a reliable and reproducible experimental tool for future research on muscle development, genetic regulation, and meat quality traits in Junmu No.1 pigs, and may also support broader applications in livestock biology and translational biomedical studies.

## 1. Introduction

Skeletal muscle tissue is the most abundant tissue in the body, accounting for approximately 40% of an animal’s weight [1,2]. Satellite cells are important adult stem cells in mammalian skeletal muscle, responsible for muscle fiber growth, regeneration, and damage repair [3]. In agriculturally important animals (e.g., pigs, cattle, sheep, chickens), the proliferation and differentiation capacity of satellite cells are directly related to muscle development efficiency and meat production performance [4]. Isolated primary satellite cells are challenging to culture in vitro due to their tendency to undergo proliferation arrest and spontaneous differentiation, as well as their limited passage capacity. These limitations severely restrict their application in studies of muscle development mechanisms, nutritional regulation, and disease modeling [5]. The Junmu No.1 White pig is a hybrid of Belgian Seghers hybrid boars and Chinese Sanjiang hybrid sows [6], characterized by rapid growth, high lean meat percentage, outstanding feed efficiency, strong adaptability, and the ability to quickly produce high-quality commercial pigs that meet market demands when crossed with Large White pigs. A stable and functionally intact immortalized porcine satellite cell (PSC) line is therefore needed to provide a consistent genetic background and sufficient cell numbers for studying the genetic regulation of muscle development in pigs.

The construction of immortalized cell lines often relies on viral-derived oncogenes (such as SV40 large T antigen/SV40LT), telomerase activation (hTERT) or spontaneous mutations leading to immortalization [7]. In bovine intestinal epithelial cells, hTERT-based immortalization successfully preserved barrier function and pathogen responsiveness [2]. SV40LT-mediated immortalization technology has also been widely used in human, mouse myocytes, and porcine skeletal muscle satellite cell research [8]. The successful establishment of immortalized satellite cell lines from Western commercial pigs [8], together with the generation of a porcine enteric smooth muscle cell line via lentiviral transduction of SV40 large T antigen [9], strongly supports the feasibility and validity of our technical strategy for immortalizing Junmu No.1 white pig satellite cells.

Despite the well-established utility of immortalized cell lines in biomedical research, relatively few immortalized satellite cell lines have been generated from livestock species, particularly pigs (Table 1). The murine C2C12 line, spontaneously immortalized from dystrophic mouse satellite cells, has served as the predominant in vitro model for myogenesis studies for over four decades [10]. In porcine species, Jiang et al. established an SV40LT-immortalized enteric smooth muscle cell line from Yorkshire pigs [9], and Ni et al. recently generated an immortalized skeletal muscle satellite cell line from Large White pigs [8]. In bovine species, Ji et al. created SV40LT-immortalized rumen epithelial cells [2], while Stout et al. employed a dual bTERT/CDK4 strategy to immortalize bovine satellite cells for cultured meat applications [11]. Notably, no immortalized satellite cell lines have been established from Chinese indigenous pig breeds, which possess distinct genetic backgrounds and muscle development characteristics compared to Western commercial breeds. The Junmu No.1 pig, a hybrid derived from Belgian Seghers hybrid boars and Chinese Sanjiang hybrid sows [6], represents a valuable genetic resource for which, to our knowledge, no immortalized in vitro satellite cell model has yet been reported.

This study aims to establish an immortalized porcine satellite cell line from Junmu No.1 pigs (imPSC-JM) through lentivirus-mediated stable transfection of the SV40LT gene. We will then systematically evaluate its proliferative characteristics, stem cell marker expression, and myogenic differentiation potential through both qualitative and quantitative comparisons with primary PSCs. The ultimate goal is to create a genetically stable, long-term passaging PSC model that can provide a continuous and reliable in vitro research resource for studying the genetic mechanisms underlying muscle development in our specific pig breed.

## 2. Materials and Methods

### 2.1. Cell Isolation and Culture

All animal procedures in this study were reviewed and approved by the Institutional Animal Care and Use Committee (IACUC) of Jilin University (Approval No. SY202506050). All procedures were performed in strict accordance with the Guidelines for the Welfare and Ethical Review of Laboratory Animals (GB/T 35892-2018) of the People’s Republic of China and the Guide for the Care and Use of Laboratory Animals (8th edition) of the U.S. National Research Council. The study adhered to the 3R principle (Replacement, Reduction, Refinement) to minimize animal suffering and the number of animals used. A two-day-old male Junmu No.1 White pig was selected for satellite cell isolation based on the following considerations. Neonatal piglets (1–3 days old) possess a high proportion of Pax7-positive satellite cells relative to total myonuclei, as satellite cell abundance declines rapidly during postnatal muscle maturation [12]. This developmental stage provides an optimal balance between cell yield and proliferative capacity, as satellite cells from neonatal animals exhibit higher proliferative potential compared to those isolated from older animals [13]. Similar age selections have been employed in previous porcine satellite cell studies: Ni et al. used 3-day-old Large White piglets [8], and Wang et al. isolated satellite cells from newborn piglets [5]. Our choice of 2-day-old animals is consistent with these established protocols and ensures maximal satellite cell yield prior to the onset of significant postnatal quiescence.

The selected piglet was euthanized at the Jilin University Agricultural Experiment Base following humane slaughter practices, and the *musculus longissimus dorsi* was collected. The back skin was incised along the ribs, and the longissimus dorsi muscles on both sides of the spine were removed. Fascia and fat were carefully trimmed away. The muscles were washed 3–5 times with pre-chilled PBS buffer supplemented with 5% antibiotic–antimycotic (Thermo Fisher Scientific, Waltham, MA, USA, 15240062) until no blood was visible. The longissimus dorsi muscle was cut into small pieces and digested in 0.2% type II collagenase (Thermo Fisher Scientific, USA, 17101015) in a shaking water bath at 37 °C for 2 h. The supernatant cell suspension was washed with Dulbecco’s modified eagle medium (DMEM) high glucose medium (Servicebio, Wuhan, China, G4511) supplemented with 1% antibiotic–antimycotic, and then sequentially filtered through 100 μm, 70 μm, and 40 μm filters (BD (Becton Dickinson), Franklin Lakes, NJ, USA, 352360, 352350, 352340) to remove tissue debris. The cells were resuspended in DMEM/F-12 medium (Servicebio, China, G4610) supplemented with 15% FBS (Pricella, Wuhan, China, 164210-50), 1% non-essential amino acids (Pricella, PB180424), 1% GlutaMax (Thermo Fisher Scientific, USA, 35050061), 1% antibiotic–antimycotic, and 2.5 ng/mL thermally stable recombinant human bFGF/FGF-2 protein (Yeasen, Shanghai, China, 91334ES60). The mixed cells were cultured in a culture dish for 2 h to remove fibroblasts by differential adhesion. All culture plates and flasks used in this study were pre-coated with Matrigel (BD, 356234) overnight at 4 °C prior to cell seeding. The purified satellite cells were transferred to new culture flasks for proliferation culture. At ~70% confluency, the proliferation medium was replaced with differentiation medium supplemented with 2% horse serum (Thermo Fisher Scientific, USA, 26050070) and 1% antibiotic–antimycotic to induce PSC to differentiate. The state of induced differentiation was observed from 24 h to 96 h.

### 2.2. Initial PSC Characterization

To assess proliferating PSC, the p2 PSC were seeded at a density of 5 × 10^4^ cells/well in 24-well plates and cultured to 70% confluency, fixed in 4% paraformaldehyde (Beyotime, Shanghai, China, P0099) for 30 min, washed three times with DPBS, permeabilized with permeabilization buffer (Beyotime, China, P0096) for 30 min, washed three times with wash buffer (Beyotime, China, P0106), and blocked with blocking buffer (Beyotime, China, P0260) at 37 °C for 1 h. Primary antibodies against PAX7 (paired box 7) and MYOD1 (myogenic differentiation 1) were diluted 1:200 in blocking buffer and incubated with the cells overnight at 4 °C. The next day, the cells were washed three times with PBST and incubated for 1 h at room temperature with secondary antibodies (Proteintech #RGAR002 for PAX7, 1:1000; Proteintech #RGAM004 for MYOD1, 1:1000) and DAPI (4’,6-diamidino-2-phenylindole) nuclear stain (Abcam #ab104139, Cambridge, UK; 1:1000) diluted in blocking buffer. Finally, the cells were washed three times and imaged using a Zeiss fluorescence microscope (Axio Vert.A1, Carl Zeiss AG, Oberkochen, Germany). All antibody information is provided in Supplementary File S2.

### 2.3. Plasmid Construction and Lentiviral Packaging

The Large T antigen gene (SV40LT, Gene ID: 29031019) was obtained from the pHAGE2-TetOminiCMV-SV40LT plasmid ( Addgene#136616, Watertown, MA, USA) by high-fidelity PCR. The SV40LT gene was inserted into a lentiviral vector derived in our laboratory by modifying pHAGE-EF1α-eGFP-W (Addgene #126686). The newly constructed plasmid contains SV40LT and the BleoR gene, encoding zeocin resistance for selection after viral transduction, separated by P2A (Porcine teschovirus 2A self-cleaving peptides) and T2A (Thosea asigna virus 2A self-cleaving peptides) peptide sequences, respectively. The plasmid was named pHAGE-EF1α-eGFP-SV40LT-BleoR and sequence is available in the Supplementary File S1. All cloning steps were performed using homologous recombination, including fragment amplification with PrimeSTAR Max DNA Polymerase Ver.2 (Takara Bio, Kusatsu, Japan, #R047), PCR purification (Promega, Madison, WI, USA, A9282), In-Fusion assembly (Takara Bio, Japan, 639649), and transformation into chemically competent *E. coli* (Takara Bio, Japan, 9057), all according to the manufacturers’ instructions. The assembly was verified by Sanger sequencing (Sangon Biotech, Shanghai, China), and the plasmid was purified at a large scale using a TIANGEN EndoFree Maxi Plasmid Kit (Tiangen, Beijing, China, DP117).

Lentiviral vectors were produced using the third-generation packaging system in 293T cells (ATCC, CRL-3216). Cells were seeded at a density of 6 × 10^6^ cells per 10 cm dish in DMEM supplemented with 10% fetal bovine serum (FBS) and 1% penicillin-streptomycin. When cells reached 70–80% confluence, they were transfected with the transfer plasmid encoding the gene of interest, along with the packaging plasmids pMDLg/pRRE, pRSV-Rev, and the envelope plasmid pMD2.G, at a ratio of 4:2:1:1 (*w*/*w*), using polyethyleneimine (PEI max, Polyscience, Warrington, PA, USA, 24765).

The transfection mix was prepared in Opti-MEM (Thermo Fisher Scientific, USA, 51985034) and incubated for 20 min at room temperature before adding to the cells. Culture supernatants containing lentiviral particles were harvested 48 h and 72 h post-transfection and filtered through a 0.45 µm filter to remove cell debris. Viral titers were determined by transducing HEK293T cells and analyzing GFP expression 72 h post-infection by flow cytometry (Sony, Tokyo, Japan, SA3800). The virus was then diluted to 10^6^ IU/mL in culture medium and stored at −80 degrees Celsius for future use. All experiments were performed under biosafety level 2 (BSL-2) conditions in accordance with institutional guidelines.

### 2.4. Cell Transfection and Screening

PSCs were detached using 0.25% Trypsin–EDTA (Thermo Fisher Scientific, USA, 25200056) and seeded at a density of 5 × 10^5^ cells/well in 6-well plates. To determine the optimal Zeocin concentration for stable cell selection, cells at approximately 50% confluency were cultured in growth medium supplemented with Zeocin (Beyotime, China, ST1450) at final concentrations of 50, 100, 150, 200, 250 or 300 μg/mL. The medium was replaced daily for three consecutive days, and 200 μg/mL Zeocin, identified as the lowest concentration resulting in complete cell death, was selected and used for all subsequent experiments.

For lentiviral transduction, cells were seeded at a density of 5 × 10^5^ cells/well in 6-well plates and cultured to approximately 50% confluency. The medium was then replaced with fresh growth medium containing 10 μg/mL Polybrene (Beyotime, China, C0351) and Lenti-EGFP-SV40LT virus at a final titer of 1 × 10^6^ IU/mL. This lentiviral vector encodes the SV40 large T antigen and a Zeocin resistance gene. After overnight incubation, the viral supernatant was replaced with fresh growth medium.

At 72 h post-transduction, cells were subjected to selection using growth medium supplemented with 200 μg/mL Zeocin. Selection was maintained for 7 days, with medium changes every 2 days, until all non-transduced cells were eliminated.

### 2.5. Long-Term Growth Curve Determination

Once the engineered cells were stably growing under Zeocin selection for 10 passages, long-term growth was assessed through serial passaging analysis. Briefly, both imPSC-JM and primary PSC were seeded in triplicate at a density of 5 × 10^5^ cells/well in 6-well plates. The culture medium was replaced every other day until the cells reached approximately 80% confluency, at which point the cells were harvested, counted using an automated cell counter (Monwei, Shanghai, China), and re-plated into new 6-well plates as described above. Every 5 passages, a sub-population of cells was cryopreserved in FBS + 10% DMSO. Primary PSC cultures were terminated after a sharp decline in cell growth, indicating the onset of senescence. The engineered cells were designated as immortalized porcine satellite cells (imPSC-JM) after achieving 110 cumulative population doublings.

### 2.6. Immunofluorescence and Analysis of imPSC-JM

imPSC-JM at passage 30 (P30) were characterized and compared with primary PSCs at passage 2 (P2) to assess the myogenic differentiation capacity of imPSC-JM. Briefly, proliferating cells were seeded at a density of 5 × 10^4^ cells/well in 24-well plates, cultured to approximately 70–80% confluence, and then fixed and stained for DAPI, PAX7, and MYOD1. For differentiation analysis, cells were seeded at a density of 5 × 10^4^ cells/well in 24-well plates, cultured to approximately 80% confluence, and switched to differentiation medium (DMEM supplemented with 2% horse serum). At 24, 48, 72, and 96 h post-induction, cells were fixed and immunostained for MYHC with DAPI counterstaining. Additional markers including PAX7, MYOD1, Desmin, and DMD were assessed at 96 h to confirm terminal differentiation. All antibody information is provided in Supplementary File S2.

For morphometric quantification of myogenic differentiation, three independent replicates were performed per group at each time point, and images were captured using a Zeiss fluorescence microscope (Axio Vert A1). The following parameters were measured using ImageJ software (version 1.54p, NIH, Bethesda, MD, USA): fusion index (percentage of nuclei within MYHC-positive myotubes containing ≥3 nuclei relative to total nuclei), average number of nuclei per myotube (counted only in MYHC-positive structures containing ≥3 nuclei), myotube length (longest axis of MYHC-positive structures containing ≥3 nuclei), myotube diameter (perpendicular to the longest axis at the widest point of MYHC-positive structures containing ≥3 nuclei), and MYHC-positive coverage. For MYHC-positive coverage quantification, MYHC fluorescence images were first subjected to threshold segmentation to generate binary masks of all MYHC-positive regions; subsequently, only MYHC-positive regions overlapping with ≥3 DAPI-stained nuclei were retained, and the coverage was calculated as the percentage of these qualified MYHC-positive areas relative to the total image area. Statistical comparisons between primary PSC and imPSC-JM at each time point were performed using multiple unpaired *t*-tests in GraphPad Prism (version 9.0), with statistical significance set at *p* < 0.05.

### 2.7. Karyotype Analysis

Karyotype analysis of imPSC-JM was performed by Sangon Biotech (Shanghai, China) at passage 20. Twenty G-banded metaphase spreads were analyzed using the Giemsa-trypsin-Giemsa (GTG) Banding technique.

### 2.8. Western Blot Analysis

The imPSC-JM were collected and lysed with RIPA lysis buffer (Beyotime, China, P0013C) containing protease inhibitor cocktail (MedChemExpress, Monmouth Junction, NJ, USA, HY-K0010) and phosphatase inhibitor cocktail (Target Molecule Corp., Boston, MA, USA, C0057) for protein extraction. The cell lysates were centrifuged (13,000× *g*, 4 °C) for 10 min, and the supernatants were collected. The protein concentrations were determined by a BCA protein assay kit (Beyotime, China, P0009). A total of 10 μg of each prepared protein sample was diluted with SDS-containing sample buffer and separated by 10% SDS-PAGE. The proteins that had been electrophoretically separated on SDS gels were transferred to PVDF (0.45 μm) membranes. Nonspecific reactivity was blocked for 1.5 h at RT (25 °C) in standard 1 × TBST buffer containing 5% BSA. The membranes were incubated overnight at 4 °C with a 1:500 dilution of anti-SV40 T antigen–antibody (MedChemExpress, USA, HY-P83511) and with a 1:8000 dilution of the anti-GAPDH antibody (Proteintech, China, 60004-1-Ig). Then, the membranes were washed for 10 min (4 times) with TBST (Servicebio, China, G0004). Subsequently, they were incubated for 1 h at room temperature with HRP-conjugated Goat Anti-Rabbit IgG(H+L) (Proteintech, China, SA00001-2) diluted 1:20,000. Immunoreactive bands were detected using an ECL Advanced Western Blotting Detection Kit (Invitrogen, Carlsbad, CA, USA, 32106), and the signal intensities were determined with the Tanon 5200 (Tanon Science & Technology Co., Ltd., Shanghai, China) chemiluminescence/fluorescence image analysis system.

### 2.9. Library Preparation and Next-Generation Sequencing

RNA sequencing (RNA-seq) was performed by Novogene Corporation (Tianjin, China). Briefly, RNA-seq was conducted on the Illumina NovaSeq platform to profile mRNAs and lncRNAs in primary PSCs and imPSC-JM. Total RNA was extracted from primary PSCs and imPSC-JM (*n* = 3 each) using the RNAprep Pure Cell/Bacteria Kit (Tiangen, Beijing, China, DP430) according to the manufacturer’s instructions. RNA quality (including integrity, RIN value) and concentration were determined using an Agilent 2100 Bioanalyzer (Agilent Technologies, Santa Clara, CA, USA). The following steps were all performed according to the manufacturer’s recommendations: rRNA removal, fragmentation, first- and second-strand cDNA synthesis, cDNA purification, terminal repair, A-tailing, adapter ligation, size selection (370–450 bp), and PCR enrichment (10 cycles). The PCR products were purified using the AMPure XP system. After library construction, preliminary quantification was carried out using Qubit, and the library’s insert fragment size was verified using an Agilent Bioanalyzer.

RNA sequencing data quality control and analysis were performed as described before [14]. Briefly, sequencing reads were filtered to remove sequences containing adapter contamination, low-quality reads, and those with a high proportion of N bases. Trimmed reads were then mapped to the pig reference genome assembly (https://ftp.ensembl.org/pub/release-114/fasta/sus_scrofa/dna/Sus_scrofa.Sscrofa11.1.dna.toplevel.fa.gz, accessed on 15 May 2025) using HISAT2 and StringTie [15]. Sequencing read alignments were further analyzed using Cufflinks/Cuffmerge to filter out reads mapped to unannotated genes and long noncoding RNAs [16]. Gene expression level was calculated as Transcripts Per Million (TPM).

### 2.10. RNA-Seq Analysis

Raw gene count files were used for Pearson correlation coefficient analysis, principal component analysis (PCA), and DESeq2 analysis. Genes with *p*adj < 0.01 and |log_2_FoldChange| > 2 were considered differentially expressed at the genome-wide level. For the targeted analysis of myogenic and pathway-specific genes (Table 2 and Table 3), a relaxed cutoff of *p*adj < 0.05 and |log_2_FoldChange| > 1 was applied.

Normalized TPM values were used to compare gene expression patterns between imPSC-JM and primary PSC. Log_10_(TPM + 1) transformed data was used to construct a linear regression model to assess consistency: PSC_TPM = β_0_ + β_1_ × imPSC_TPM + ε. High consistency between the two datasets was inferred if the slope β_1_ ≈ 1 and the intercept β_0_ ≈ 0, where ε represents the random error. The model fit was evaluated using R^2^.

We generated clustering heatmaps of the two datasets to visualize gene expression patterns. In addition to a heatmap of all genes, we downloaded muscle development-related Gene Ontology (GO) terms from the GO database and created a new gene set (Supplementary File S3) comprising all genes associated with these terms [17]. We then generated a separate heatmap displaying the expression levels of this muscle development gene set.

For targeted analysis of myogenic gene expression, key genes involved in satellite cell identity (PAX7, PAX3), myogenic regulatory factors (MYOD1, MYF5, MYOG, MYF6), muscle structural components (MYH1, MYH4, MYH7, DES, DMD, ACTA1), satellite cell regulation (SPRY1, HES1), myoblast fusion (MYMK, MYMX), and muscle signaling (MSTN, FOXO1) were extracted from the TPM dataset. Mean TPM values were calculated for each group, and log2 fold changes were computed as log_2_[(mean_imPSC + 1)/(mean_PSC + 1)]. Statistical significance was assessed using Student’s *t*-test with Benjamini–Hochberg correction for multiple comparisons. Gene Set Enrichment Analysis (GSEA) was conducted using the clusterProfiler package (version 4.2.0) with a pig KEGG pathway gene set database as the background. Pathways with |normalized enrichment score (NES)| > 2, *p* < 0.01, and false discovery rate (FDR) < 0.05 were considered significantly enriched. To validate GSEA findings at the individual gene level, key genes within significantly enriched pathways (autophagy, inflammatory signaling, and ribosome biogenesis) were extracted and their differential expression was analyzed using the same statistical approach as described above.

All analyses were performed using R v4.5.0.

### 2.11. Quantitative Real-Time PCR (qPCR)

Total RNA was extracted from primary PSC and imPSC-JM using the RNAprep Pure Cell/Bacteria Kit (DP430, Tiangen, China) according to the manufacturer’s instructions. The quality and concentration of the RNA were detected with a spectrophotometer (Nanodrop 2000, Thermo Scientific, USA). Next, cDNA was synthesized by reverse transcription using the PrimeScript™ RT Reagent Kit with gDNA Eraser (R047A, TaKaRa, Japan) according to the manufacturer’s instructions. qPCR was performed on a PCRmax Eco48 instrument (PCRmax, Altrincham, UK) using SYBR Green Master Mix (Roche, Indianapolis, IN, USA, 04913914001). The primers used for qPCR were designed with NCBI Primer Blast. The qPCR primers for each gene are shown in Supplement File S4. The relative mRNA expression levels were determined using the 2^−ΔΔCt^ method.

## 3. Results

### 3.1. Isolation and Morphological Characterization of Primary PSCs

**Figure 1:**
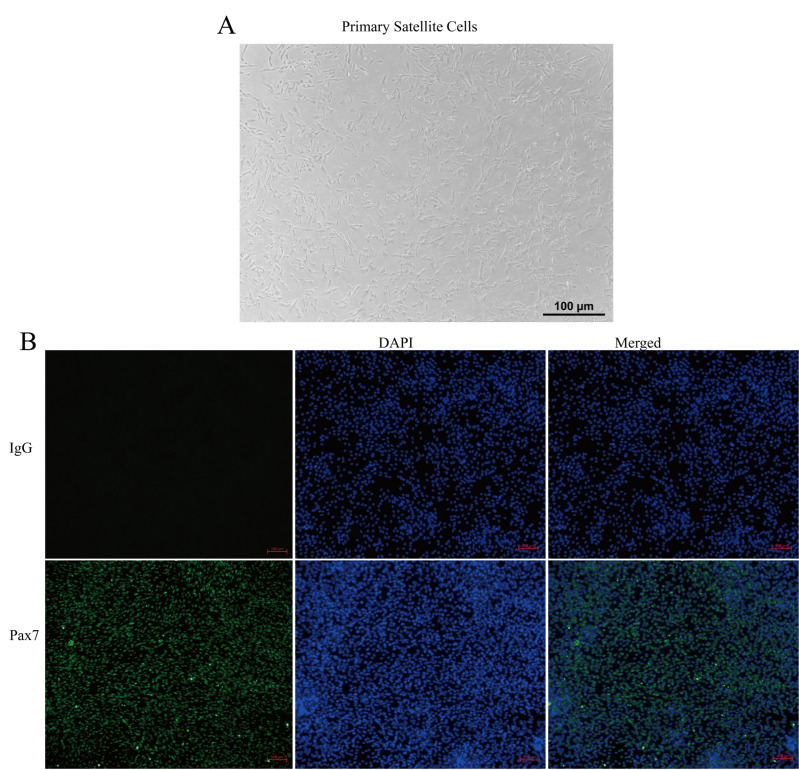
Isolation and identification of primary porcine satellite cells (PSCs). (**A**) Phase-contrast image of primary PSCs showing typical spindle-shaped morphology. Scale bar, 100 μm. (**B**) Immunofluorescence staining of PAX7 (green) with DAPI (blue) counterstaining; isotype-matched IgG was used as a negative control. The vast majority of cells were PAX7-positive. Scale bars, 100 μm.

Primary PSCs were isolated from the longissimus dorsi muscle of a 2-day-old Junmu No.1 piglet using enzymatic digestion combined with differential adhesion. Phase-contrast microscopy showed that the adherent cells exhibited a uniform, spindle-shaped, myoblast-like morphology (Figure 1A). Immunofluorescence staining demonstrated that the vast majority of cells were positive for the satellite cell-specific marker PAX7 (green), with nuclei counterstained by DAPI (blue), while no signal was detected in the isotype-matched IgG negative control (Figure 1B). These results confirm that the isolated cells were skeletal muscle satellite cells suitable for subsequent immortalization.

### 3.2. Establishment of the Immortalized Porcine Satellite Cell Line (imPSC-JM)

**Figure 2:**
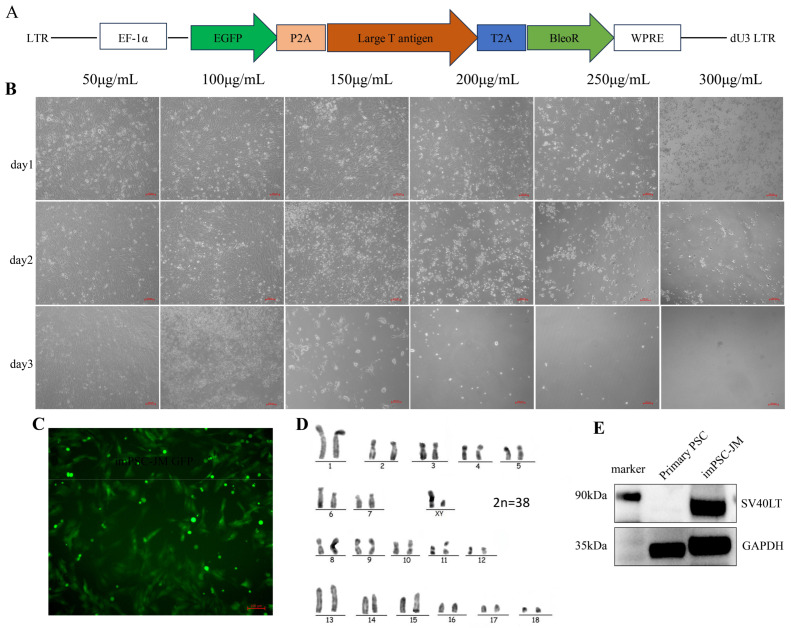
Establishment of the immortalized porcine satellite cell line (imPSC-JM). (**A**) Schematic of the lentiviral vector pHAGE-EF1α-eGFP-SV40LT-BleoR. (**B**) Zeocin kill curve of primary PSCs (50–300 μg/mL, days 1–3); 200 μg/mL was selected as the working concentration. Scale bars, 100 μm. (**C**) EGFP fluorescence in Zeocin-selected imPSC-JM. Scale bar, 100 μm. (**D**) G-banded karyotype of imPSC-JM at passage 20 showing a normal diploid porcine karyotype (2*n* = 38, XY). (**E**) Western blot of SV40LT (~90 kDa) in primary PSCs and imPSC-JM, with GAPDH (~35 kDa) as loading control. (the original Western blot pictures can be found in Supplementary File S5).

Primary PSCs were transduced with the SV40LT-encoding lentivirus (Figure 2A; Section 2.3 and Section 2.4) and selected with Zeocin. The Zeocin kill curve showed complete elimination of non-transduced cells at 200 μg/mL Zeocin by day 3 (Figure 2B).

Following lentiviral transduction and Zeocin selection, fluorescence microscopy showed that the vast majority of surviving cells expressed EGFP (Figure 2C), indicating efficient lentiviral integration and transgene expression. Western blot analysis detected a specific SV40LT band at ~90 kDa in imPSC-JM but not in primary PSCs, with GAPDH (~35 kDa) used as a loading control (Figure 2E), confirming stable integration and expression of the SV40LT transgene. Karyotype analysis further demonstrated that imPSC-JM retained a normal diploid porcine karyotype (2*n* = 38, XY) (Figure 2D), indicating that the immortalization procedure did not induce gross chromosomal aneuploidy. Together, these results confirm the successful establishment of an EGFP- and SV40LT-expressing immortalized porcine satellite cell line, designated imPSC-JM, with a stable karyotype.

### 3.3. imPSC-JM Exhibits Long-Term Proliferative Capacity and Retains Myogenic Identity

**Figure 3:**
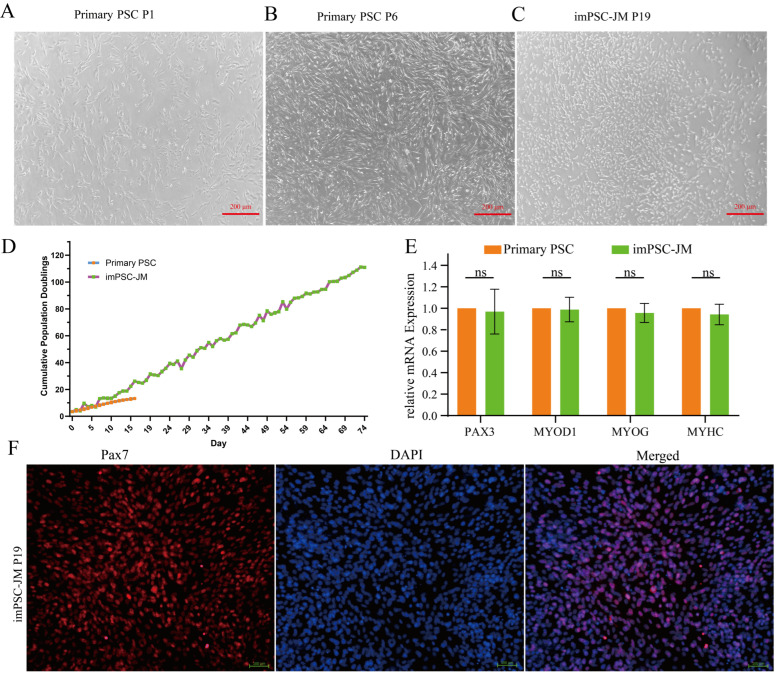
Long-term proliferation and retention of myogenic identity in imPSC-JM. (**A**–**C**) Phase-contrast images of primary PSCs at P1 (**A**) and P6 (**B**), and imPSC-JM at P19 (**C**). Scale bars, 200 μm. (**D**) Cumulative population doublings (CPDs) of primary PSCs (orange) and imPSC-JM (green); primary PSCs ceased proliferation after ~14 CPDs, whereas imPSC-JM exceeded 110 CPDs over 74 days. (**E**) RT-qPCR analysis of *PAX3*, *MYOD1*, *MYOG*, and *MYHC*. Mean ± SD (*n* = 3); Student’s *t*-test; ns, not significant. (**F**) Immunofluorescence of PAX7 (red) and DAPI (blue) in imPSC-JM at P19. Scale bars, 100 μm.

To evaluate the proliferative capacity and phenotypic stability of the immortalized cell line, primary PSCs and imPSC-JM were continuously passaged and compared. Phase-contrast microscopy showed that primary PSCs at passage 1 (P1) displayed a typical spindle-shaped, myoblast-like morphology with good adherence (Figure 3A), but by passage 6 (P6) they became flattened and enlarged, exhibiting senescence-associated morphology (Figure 3B). In contrast, imPSC-JM at passage 19 (P19) retained a uniform spindle-shaped morphology without signs of senescence (Figure 3C). Cumulative population doubling (CPD) analysis further demonstrated that primary PSCs ceased to proliferate after approximately 16 days (~14 CPDs), whereas imPSC-JM proliferated continuously for more than 74 days, reaching over 110 CPDs (Figure 3D). These results indicate that imPSC-JM bypasses replicative senescence and possesses a robust long-term proliferative capacity.

To assess whether immortalization affected the myogenic identity of the cells, RT-qPCR was performed to examine the expression of PAX3, MYOD1, MYOG, and MYHC in primary PSCs and imPSC-JM. No significant differences were detected between the two groups for any of the four genes (*n* = 3, ns; Figure 3E). Immunofluorescence staining of imPSC-JM at P19 further showed that the vast majority of cells remained PAX7-positive, with nuclei counterstained by DAPI (Figure 3F). These results demonstrate that imPSC-JM retains the myogenic gene expression profile and the satellite-cell identity of primary PSCs after long-term passaging.

### 3.4. imPSC-JM Retains Myogenic Differentiation Capacity with Reduced Fusion Efficiency

**Figure 4:**
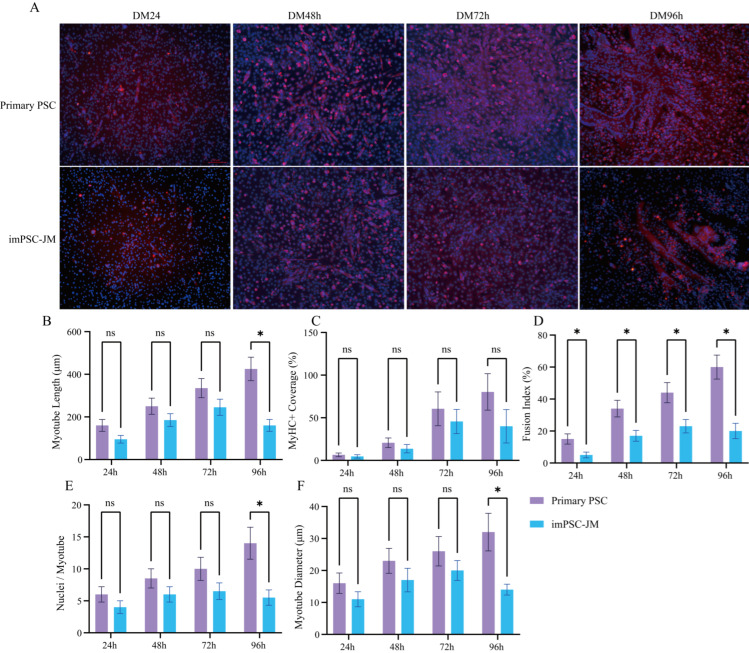
Myogenic differentiation of primary PSCs and imPSC-JM. (**A**) MYHC (red) and DAPI (blue) staining of primary PSCs (top) and imPSC-JM (bottom) at 24, 48, 72, and 96 h in differentiation medium. Scale bars, 100 μm. (**B**–**F**) Quantification of (**B**) fusion index, (**C**) myotube length, (**D**) myotube diameter, (**E**) nuclei per myotube, and (**F**) MYHC^+^ area coverage. Mean ± SD (*n* = 3); multiple unpaired Student’s *t*-tests. * *p* < 0.05; ns, not significant.

Myogenic differentiation of imPSC-JM and primary PSCs was assessed by MYHC immunofluorescence at 24, 48, 72, and 96 h post-induction (protocol described in Section 2.6). Both cell types formed multinucleated MYHC^+^ myotubes at all time points examined, with the number, length, and multinucleation of myotubes progressively increasing over time (Figure 4A). However, imPSC-JM consistently produced fewer, shorter, and less multinucleated myotubes than primary PSCs, indicating that the differentiation capacity was preserved but with reduced efficiency.

Quantitative analyses further confirmed these observations. The fusion index (Figure 4B), myotube length (Figure 4C), and number of nuclei per myotube (Figure 4E) of imPSC-JM were significantly lower than those of primary PSCs at all four time points (*p* < 0.05). Myotube diameter showed no significant difference at 24, 48, or 72 h, but became significantly smaller in imPSC-JM at 96 h (Figure 4D). The MYHC^+^ area coverage exhibited a consistent trend toward lower values in imPSC-JM compared with primary PSCs, although the differences did not reach statistical significance at any time point (Figure 4F). Together, these results demonstrate that imPSC-JM retains the capacity to undergo myogenic differentiation and to form multinucleated myotubes, but with a reduced differentiation efficiency relative to primary PSCs.

### 3.5. Comparative Transcriptomic Analysis Reveals Global Similarity and Specific Pathway Remodeling Between Primary PSCs and imPSC-JM

**Figure 5:**
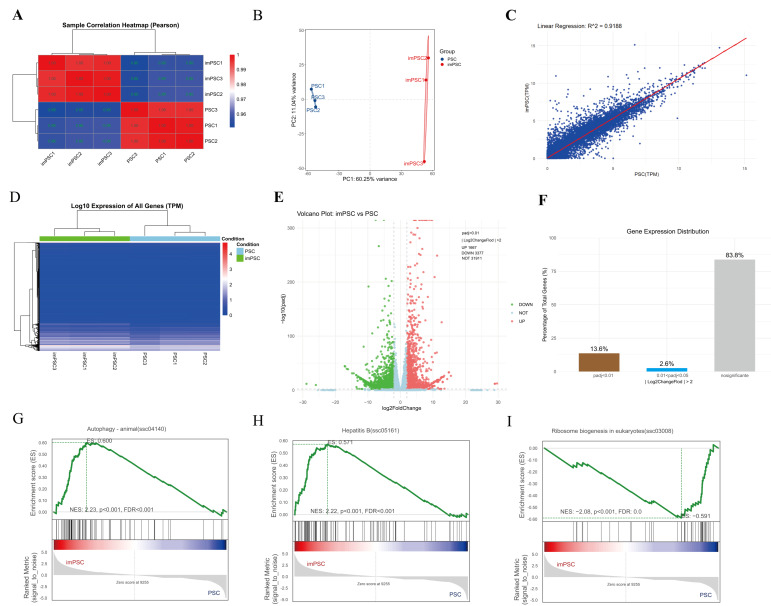
Transcriptomic comparison between primary PSCs and imPSC-JM (*n* = 3 each). (**A**) Pearson correlation heatmap (intra-group r > 0.99; inter-group r ≥ 0.95). (**B**) PCA plot (PC1, 60.25%; PC2, 11.04%). (**C**) Linear regression of all-gene TPM values (R^2^ = 0.9188). (**D**) Hierarchical clustering heatmap of log_10_(TPM) for all expressed genes. (**E**) Volcano plot of differentially expressed genes (*p*adj < 0.01, |log_2_FC| > 2): 1667 upregulated and 3377 downregulated in imPSC-JM. (**F**) Proportion of genes by significance: 83.8% unchanged, 13.6% *p*adj < 0.01, and 2.6% with 0.01 < *p*adj < 0.05. (**G**–**I**) KEGG GSEA showing upregulation of (**G**) Autophagy-animal (NES = 2.23) and (**H**) Hepatitis B (NES = 2.22), and downregulation of (**I**) Ribosome biogenesis in eukaryotes (NES = −2.08); all *p* < 0.001, FDR < 0.05.

To systematically compare the molecular profiles of primary PSCs and imPSC-JM, RNA sequencing was performed on three biological replicates of each cell type. Pearson correlation analysis revealed high intra-group consistency (r > 0.99) and strong inter-group correlation (Pearson r ≥ 0.95), with hierarchical clustering clearly separating the two cell types into distinct branches (Figure 5A). Principal component analysis (PCA) further revealed that PC1 accounted for 60.25% of the total variance and fully separated imPSC-JM from primary PSCs, while PC2 explained 11.04% of the variance; samples within each group clustered tightly, confirming good replicate consistency (Figure 5B). Genome-wide TPM scatter plot analysis demonstrated a strong linear correlation between the two populations (R^2^ = 0.9188; Figure 5C), and hierarchical clustering of log_10_-transformed TPM values of all genes consistently grouped imPSC-JM and PSC samples into two separate branches (Figure 5D). Together, these results indicate that imPSC-JM largely preserves the overall transcriptional profile of primary PSCs while exhibiting reproducible global differences associated with immortalization.

To identify genes underlying these differences, differential expression analysis was performed using *p*adj < 0.01 and |log_2_FoldChange| > 2 as cutoffs. A total of 1667 genes were upregulated and 3377 genes were downregulated in imPSC-JM compared with primary PSCs, while 31,911 genes (including all genes with *p*adj > 0.01) showed no significant change (Figure 5E). Overall, the strictly defined differentially expressed genes (DEGs) accounted for 13.6% of all expressed genes, genes with 0.01 < *p*adj < 0.05 accounted for 2.6%, and the remaining 83.8% of genes were not significantly altered (Figure 5F), further supporting the high transcriptomic similarity between the two cell populations.

To explore the biological pathways affected by immortalization, gene set enrichment analysis (GSEA) was performed against the KEGG database. Two pathways were significantly enriched in imPSC-JM: “Autophagy-animal” (ssc04140; NES = 2.23, *p* < 0.001, FDR < 0.001; Figure 5G) and “Hepatitis B” (ssc05161; NES = 2.22, *p* < 0.001, FDR < 0.001; Figure 5H), suggesting that immortalization upregulates autophagy-related processes and viral-infection-associated signaling cascades, consistent with the introduction of SV40LT. In contrast, the “Ribosome biogenesis in eukaryotes” pathway (ssc03008; NES = −2.08, *p* < 0.001, FDR < 0.001; Figure 5I) was significantly depleted in imPSC-JM, indicating reduced ribosomal assembly activity relative to primary PSCs. Collectively, these analyses demonstrate that imPSC-JM retains the global transcriptional architecture of primary PSCs while exhibiting selective pathway remodeling, particularly in autophagy, viral-related signaling, and ribosome biogenesis.

## 4. Discussion

In this study, we successfully established an immortalized porcine satellite cell line (imPSC-JM) from neonatal Junmu No.1 piglets via lentivirus-mediated stable transduction of the SV40 large T antigen (SV40LT). The resulting line exhibited four key features: (i) sustained proliferation exceeding 110 population doublings (PDs) without senescence; (ii) a stable diploid karyotype and characteristic spindle-shaped morphology; (iii) preservation of the canonical satellite cell marker PAX7 and full myogenic differentiation capacity, as evidenced by the formation of multinucleated myotubes expressing Desmin, MYHC, and DMD (Figure S1); and (iv) high transcriptomic fidelity to primary porcine satellite cells (PSCs) (Pearson r ≥ 0.95; R^2^ = 0.9188; 83.8% of expressed genes unaltered). To our knowledge, imPSC-JM is the first immortalized satellite cell line derived from a Chinese-developed pig breed, filling a critical gap in cellular tools for porcine myogenesis research and providing a stable, genetically defined platform for dissecting the molecular basis of the unique muscle traits of Junmu No.1 pigs.

The successful establishment of imPSC-JM extends the growing repertoire of immortalized myogenic cell lines from livestock species. C2C12, spontaneously immortalized from dystrophic mouse satellite cells nearly five decades ago, has long served as the predominant in vitro model for myogenesis [10]. However, marked interspecies differences in muscle fiber-type composition, postnatal growth kinetics, and metabolic regulation between rodents and large livestock animals limit the translational value of murine models for agriculturally relevant questions [18,19]. To overcome this limitation, immortalized satellite cell lines have recently been generated from cattle [11] and Western commercial pigs (Large White) [8], alongside immortalized epithelial and smooth muscle lines from cattle and pigs [2,9]. Notably, all previously reported porcine satellite cell lines have been derived from Western commercial breeds (e.g., Large White, Yorkshire), none of which adequately represent the genetic background of Chinese-developed breeds. The Junmu No.1 pig, a hybrid integrating Belgian Seghers and Chinese Sanjiang genetic resources, exhibits a distinctive combination of rapid growth, high lean meat percentage, and environmental adaptability that cannot be recapitulated by purely European or purely Chinese genetic backgrounds [6]. imPSC-JM thus provides a much-needed cellular platform tailored to this unique genetic resource and complements the existing portfolio of livestock myogenic lines.

imPSC-JM achieved a greater proliferative capacity (>110 CPDs) than the >P40 passaging capacity reported for the Large White-derived line by Ni et al. [8], although direct comparison is limited by the different metrics used (cumulative population doublings vs. passage number) and would require head-to-head experiments under identical conditions for confirmation. The stable diploid karyotype maintained in imPSC-JM is consistent with reports in immortalized bovine and porcine cells [2,9], reinforcing the general observation that SV40LT-mediated immortalization, when performed under optimized conditions, does not necessarily induce gross chromosomal abnormalities. Importantly, our quantitative time-course analysis of myogenic differentiation provides a level of mechanistic detail that has been lacking in previous descriptive characterizations of immortalized livestock satellite cell lines.

A central observation of our study is that imPSC-JM, while retaining the core myogenic transcriptional program, exhibits significantly reduced fusion efficiency relative to primary PSCs. Quantitative time-course analysis revealed that this reduction is highly parameter-specific: the fusion index was significantly lower at all time points (24–96 h), whereas myotube length, diameter, nuclei per myotube, and MYHC-positive coverage remained largely comparable during the early differentiation phase (24–72 h), with significant divergence emerging only at 96 h. This pattern is mechanistically interpretable in light of SV40LT biology: by inactivating the p53 and Rb tumor-suppressor pathways, SV40LT disrupts the irreversible cell-cycle exit that is a prerequisite for terminal myogenic differentiation and efficient myoblast fusion [20,21,22]. Our findings are consistent with previous observations in extensively passaged C2C12 cells [23] and other SV40LT-immortalized myogenic lines [8], collectively supporting the view that fusion efficiency is the differentiation parameter most sensitively affected by oncogene-mediated immortalization.

The dissociation we observed between fusion efficiency and MYHC-positive coverage is conceptually significant: it indicates that activation of the myogenic transcriptional program (driven by MYOD1 and MYOG and reflected in muscle structural gene expression) is largely uncoupled from the cell-fusion machinery in immortalized cells. Analogous dissociations have been documented in MYMK-deficient myoblasts [24] and in aged satellite cells [25], where myogenic gene expression is preserved but multinucleation is impaired. From a practical standpoint, this dissociation defines the appropriate use case for imPSC-JM: the line is well suited for studies of satellite cell quiescence, activation, proliferation, early myogenic commitment, and muscle-specific gene regulation, but should be supplemented with primary cells when the experimental endpoint specifically requires high-efficiency fusion or extensive myofiber maturation. Based on our time-course data, we recommend a 72 h differentiation protocol as the optimal window for most applications, since this time point captures peak myotube parameters before the onset of decline. With respect to passage-dependent phenotypic drift, continued culture of imPSC-JM beyond P50 has not revealed overt changes in PAX7 immunoreactivity, proliferation, or morphology; nevertheless, potential late-passage drift in fusion efficiency warrants formal evaluation in future work.

The remarkable transcriptomic concordance between imPSC-JM and primary PSCs (Pearson r ≥ 0.95; R^2^ = 0.9188; 83.8% of expressed genes unaltered) provides robust support for the use of imPSC-JM as a faithful surrogate for primary satellite cells in molecular and genetic studies. Targeted analysis of myogenic regulatory factors (MYOD1, MYF5, MYOG, MYF6), satellite cell identity markers (PAX7, PAX3), muscle structural genes (MYH1, MYH4, MYH7, DES, DMD, ACTA1), and fusion regulators (MYMK, MYMX) revealed no significant differences, demonstrating that the foundational myogenic transcriptional architecture is fully preserved. The moderate upregulation of SPRY1—a negative regulator of receptor tyrosine kinase signaling implicated in maintaining satellite cell quiescence [26]—may even reflect an enhanced capacity of imPSC-JM to sustain the undifferentiated state during long-term proliferation, a feature that could be advantageous for experimental applications requiring large quantities of homogeneous, uncommitted cells.

Beyond this overall fidelity, GSEA and gene-level analyses revealed reproducible pathway remodeling associated with immortalization. The coordinated upregulation of autophagy-related genes (ATG16L2, MAP1LC3A/B, CTSL) and inflammatory mediators (IL6, CXCL8, CXCL2), together with downregulation of ribosome-biogenesis components (NPM1), defines a coherent adaptive program that supports sustained proliferation. Mechanistically, the IL6–JAK–STAT3 axis has been shown to drive CTSL-mediated autophagy in cancer cells [27], suggesting that an analogous regulatory loop may operate in imPSC-JM to maintain cellular homeostasis under continuous proliferative stress. The downregulation of ribosome biogenesis is particularly noteworthy: rather than indicating a generic loss of biosynthetic capacity, it likely reflects a resource-allocation strategy that favors DNA replication, autophagic recycling, and stress-resistance pathways over de novo ribosome assembly [28,29]. Similar metabolic reprogramming has been documented in immortalized murine tenocytes [7], suggesting that this transcriptional signature may represent a general hallmark of SV40LT-mediated immortalization across cell types and species. These pathway-level insights not only validate imPSC-JM as a model system but also inform future strategies for engineering immortalized cell lines with improved fidelity.

Beyond providing a defined model for Junmu No.1 myogenesis, imPSC-JM carries broader implications for porcine biology, translational research, and cellular agriculture. As a stable platform with a fixed genetic background, the line is well positioned to dissect the molecular determinants of breed-specific traits such as fiber-type composition, lean meat deposition, and feed efficiency that underpin the agricultural value of Junmu No.1 pigs. Given the increasing recognition of pigs as a translational biomedical model [19], imPSC-JM may also serve as a physiologically more relevant alternative to murine C2C12 cells for studying human muscular dystrophies, sarcopenia, and metabolic muscle disorders, while concurrently offering a defined, scalable resource for cultured meat applications, where porcine-derived myogenic cell lines remain in short supply [30,31]. These prospects, however, must be weighed against several limitations of the present work: characterization was performed on a single clonal population from one donor, neonatal isolation may not fully recapitulate the biology of adult quiescent satellite cells, the modest reduction in fusion efficiency represents an inherent trade-off of SV40LT-mediated immortalization, and our analysis was confined to the transcriptome, leaving proteomic, epigenomic, and metabolomic remodeling unexplored. Multi-donor validation and multi-omics profiling will therefore be important next steps for establishing the generalizability of this model.

Looking forward, imPSC-JM provides an ideal substrate for systematic functional genomics, including CRISPR/Cas9-mediated knockout, knock-in, and large-scale screens, to dissect the regulatory networks underlying muscle development and meat quality in this breed. Comparative analyses with immortalized lines from other pig breeds, such as Large White, Meishan, or Bama, may further illuminate the genetic basis of breed-specific muscle phenotypes, while controlled exposure of the line to nutritional, hormonal, and environmental cues offers direct relevance to feed formulation and management strategies. In parallel, the dissociation we observed between fusion efficiency and myogenic gene expression motivates the engineering of next-generation immortalized lines—for instance, by employing inducible or reversible SV40LT systems, combining hTERT/CDK4 strategies [11], or applying small-molecule modulators of the p53/Rb pathways—to better preserve fusion competence while retaining proliferative capacity. Such refinements would expand the applicability of imPSC-JM to disease modeling of muscular dystrophy and metabolic myopathies, as well as to emerging tissue-engineering and cultured-meat platforms addressing pressing challenges in sustainable food production.

## 5. Conclusions

In summary, imPSC-JM—the first immortalized satellite cell line established from a Chinese-developed pig breed—provides a stable, genetically consistent, and transcriptomically faithful in vitro model that fills a critical gap in cellular tools for porcine muscle biology. Beyond its immediate utility for studying Junmu No.1 pigs, this resource and the mechanistic insights gained from its characterization contribute more broadly to our understanding of cellular immortalization, the principles of satellite cell biology, and the rational design of next-generation myogenic cell models for both agricultural and biomedical applications.

## Figures and Tables

**Table 1:** Summary of immortalized cell lines from livestock and model species relevant to the present study.

Reference	Species	Breed/Strain	Cell Type	Immortalization Method	Proliferative Capacity	Demonstrated Applications
Yaffe & Saxel (1977) [10]	Mouse	C3H (dystrophic)	Skeletal muscle satellite cells (C2C12)	Spontaneous (serial passaging)	Indefinite	Research on myogenesis mechanisms; drug screening; signaling pathway analysis; muscle disease models.
Jiang et al. (2020) [9]	Pig	Yorkshire	Enteric smooth muscle cells (ileum, PIC7)	SV40LT	Not reported	porcine intestinal smooth muscle physiological research model.
Ji et al. (2021) [2]	Bovine	Angus crossbred	Rumen epithelial cells (BREC1)	SV40LT	Not reported	rumen epithelial barrier function research model.
Stout et al. (2023) [11]	Bovine	Not specified	Skeletal muscle satellite cells	bTERT + CDK4	>120 PDs	Applications of cultured meat; validation of differentiation capacity
Ni et al. (2024) [8]	Pig	Large White	Skeletal muscle satellite cells	SV40LT	>P40	porcine skeletal muscle development study.

Note: PDs: population doublings; P: passage number.

**Table 2:** Expression analysis of key myogenic genes in imPSC-JM compared to primary PSC.

Gene Category	Gene Symbol	Gene Name	log_2_FC	*p*adj	Interpretation
Satellite cell markers	PAX7	Paired box 7	−0.02	0.441	ns
	PAX3	Paired box 3	NA	NA	NA
Myogenic regulatory factors	MYOD1	Myogenic differentiation 1	−0.22	0.123	ns
	MYF5	Myogenic factor 5	−0.15	0.0755	ns
	MYOG	Myogenin	−0.52	0.00151	ns
	MYF6	Myogenic factor 6 (MRF4)	−0.02	0.475	ns
Muscle structural genes	MYH1	Myosin heavy chain 1	−0.01	0.118	ns
	MYH2	Myosin heavy chain 2	NA	NA	NA
	MYH4	Myosin heavy chain 4	−0.01	0.367	ns
	MYH7	Myosin heavy chain 7	−0.02	0.367	ns
	DES	Desmin	−1.88	0.123	ns
	DMD	Dystrophin	0.8	0.123	ns
	ACTA1	Actin alpha 1	0.01	0.475	ns
	TTN	Titin	NA	NA	NA
Regulation signaling	SPRY1	Sprouty RTK signaling antagonist 1	1.38	0.0241	Up *
	NOTCH1	Notch receptor 1	NA	NA	NA
	HES1	Hes family bHLH transcription factor 1	−0.54	0.29	ns
	MYMK	Myomaker	−0.01	0.7	ns
	MYMX	Myomixer	−0.06	0.475	ns
	IGF1	Insulin-like growth factor 1	NA	NA	NA
	MSTN	Myostatin	−0.5	0.0755	ns
	FOXO1	Forkhead box O1	−1.3	0.367	ns

Note: log_2_FC: log2 fold change (imPSC-JM vs. PSC); *p*adj: Benjamini–Hochberg adjusted *p*-value. Up: significantly upregulated (*p*adj < 0.05, log_2_FC > 1); ns: not significantly altered under the relaxed cutoff (*p*adj ≥ 0.05 or |log_2_FC| ≤ 1). * SPRY1 upregulation may reflect enhanced quiescence maintenance in immortalized cells. Genes labeled “NA” were excluded due to low or undetectable expression.

**Table 3:** Key differentially expressed genes in immortalization-associated pathways.

Pathway	Gene Symbol	Function	log_2_FC	*p*adj	Interpretation
Autophagy	CTSL	Lysosomal protease	1.98	0.00171	Up
	MAP1LC3A	Autophagosome membrane component	1.21	0.0139	Up
	ATG16L2	Autophagosome formation	1.13	0.0305	Up
	MAP1LC3B	Autophagosome membrane component	1.05	0.0249	Up
Inflammatory signaling	IL6	Pro-inflammatory cytokine	6.1	0.0139	Up
	CXCL8	Chemokine (IL-8)	5.81	0.0139	Up
	CXCL2	Chemokine	1.72	0.0139	Up
Ribosome biogenesis	NPM1	Ribosome assembly	−1.4	0.0305	Down

Note: log_2_FC: log2 fold change (imPSC-JM vs. PSC); *p*adj: Benjamini–Hochberg adjusted *p*-value. Up: significantly upregulated (*p*adj < 0.05, log_2_FC > 1); Down: significantly downregulated (*p*adj < 0.05, log_2_FC < −1).

## Data Availability

The original contributions presented in this study are included in the article/Supplementary Materials. Further inquiries can be directed to the corresponding authors.

## Supplementary Materials

The following supporting information can be downloaded at: https://www.mdpi.com/article/10.3390/vetsci13060556/s1, Figure S1: Immunofluorescence staining of mature myogenic markers in differentiated imPSC-JM. Supplementary File S1: Complete sequence of the pHAGE-EF1α-eGFP-SV40LT-BleoR lentiviral vector; Supplementary File S2: Detailed antibody information used for immunofluorescence and Western blot; Supplementary File S3: Muscle development-related gene set compiled from GO terms; Supplementary File S4: qPCR primer sequences for each target gene; Supplementary File S5: Original images of Western-blotting.

## Abbreviations

The following abbreviations are used in this manuscript:

PSC	Porcine satellite cells
imPSC-JM	Immortalized porcine satellite cells from Junmu No.1
SV40LT	Simian virus 40 large T antigen
hTERT	Human telomerase reverse transcriptase
PAX7	Paired box 7
MYOD1	Myogenic differentiation 1
MYOG	Myogenin
MYHC	Myosin heavy chain
CPD	Cumulative population doubling
DEG	Differentially expressed gene
GSEA	Gene Set Enrichment Analysis
