# Supplementary material for: Establishment and Characterization of an Immortalized Porcine Satellite Cell Line from China Junmu No.1 Pigs
Source: Vet Sci. 2026 Jun 4;13(6):556. doi: 10.3390/vetsci13060556 (PMC13308346; doi:10.3390/vetsci13060556)
Supplement: Supplementary file 1 [file vetsci-13-00556-s001.zip › Figure S1 Immunofluorescence staining of mature myogenic markers in differen-tiated imPSC-JM..pdf]

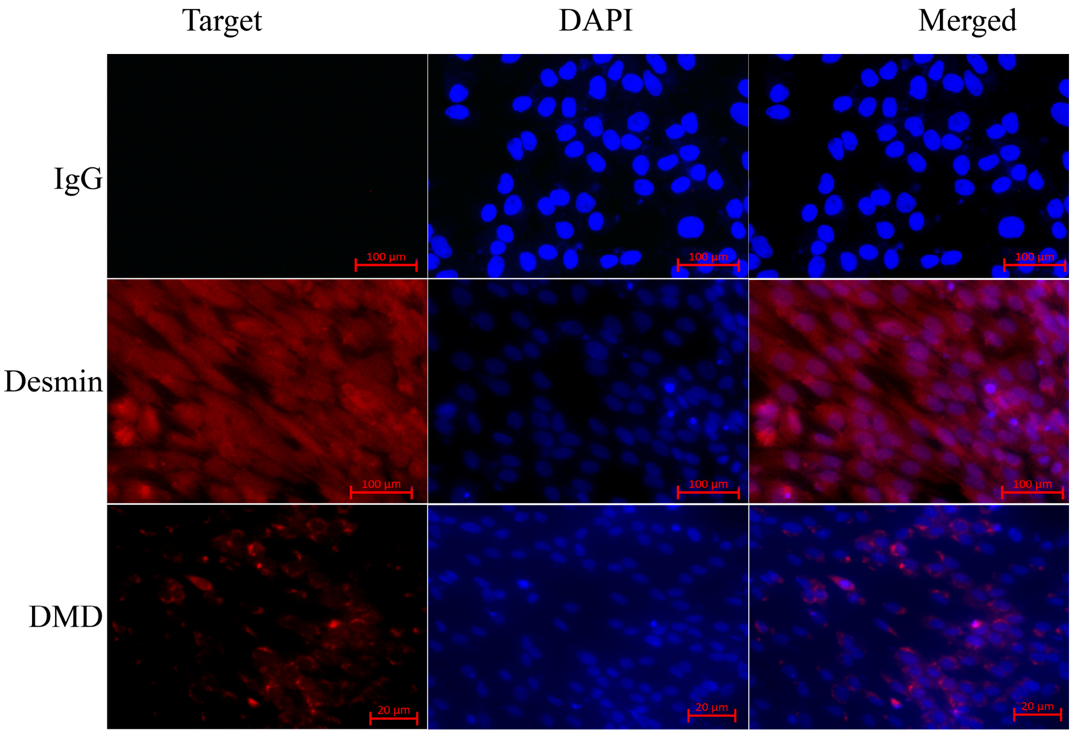

Figure S1: Immunofluorescence staining of mature myogenic markers in differen-tiated imPSC-JM. After 96 h of induction in differentiation medium, imPSC-JM were fixed and immunostained for Desmin (red, middle row) and Dystrophin (DMD, red, bottom row), with nuclei counterstained by DAPI (blue). An isotype-matched IgG was used as a negative control (top row), showing no detectable red fluorescence signal. Both Desmin and DMD were robustly expressed in differentiated imPSC-JM, confirm-ing successful terminal myogenic differentiation and the formation of mature mus-cle-specific cytoskeletal and membrane-associated structures. Scale bars: 100 µm (IgG and Desmin panels); 20 µm (DMD panels).
