# Supplementary material for: Establishment and Characterization of an Immortalized Porcine Satellite Cell Line from China Junmu No.1 Pigs
Source: Vet Sci. 2026 Jun 4;13(6):556. doi: 10.3390/vetsci13060556 (PMC13308346; doi:10.3390/vetsci13060556)
Supplement: Supplementary file 1 [file vetsci-13-00556-s001.zip › Supplementary File S1_pHAGE-EF1a-EGFP-large T antigen.seq.pdf]

Supplement File S1. The plasmid sequence of pHAGE-EF1aL-eGFP-SV40LT-BleoR

> pHAGE-EF1aL-eGFP-SV40LT-BleoR

TGTACAAGGGATCTGGAGCAACAAACTTCTCACTACTCAAACAAGCAGGTG
ACGTGGAGGAGAATCCCGGGCCTTCTAGAATGGATAAAGTTTTAAACAGAG
AGGAATCTTTGCAGCTAATGGACCTTCTAGGTCTTGAAAGGAGTGCCTGGGG
GAATATTCCTCTGATGAGAAAGGCATATTTAAAAAAATGCAAGGAGTTTCATC
CTGATAAAGGAGGAGATGAAGAAAAAATGAAGAAAATGAATACTCTGTACA
AGAAAATGGAAGATGGAGTAAAATATGCTCATCAACCTGACTTTGGAGGCTT
CTGGGATGCAACTGAGGTATTTGCTTCTTCCTTAAATCCTGGTGTTGATGCAA
TATACTGCAAACAATGGCCTGAGTGTGTAAAGAAAATGTCTACTAACTGCAT
ATGCTTGCTGTGCTTACTGAGGATGAAGCATGAAAATAGAAAATTATACAGG
AAAGATCCACTTGTGTGGGTTGATTGTTACTGCTTCGATTGCTTTAGAATGTG
GTTTGGACTTGATCTTTGTGAAGGAACCTTACTTCTGTGGTGTGACATAATTG
GACAAACTACCTACAGAGATTTAAAGCTCTAAGGTAAATATAAAATTTTTAAG
TGTATAATGTGTTAAACTACTGATTCTAATTGTTTGTGTATTTTAGATTCCAAC
CTATGGAACTGATGAATGGGAGCAGTGGTGGAATGCCTTTAATGAGGAAAAC
CTGTTTTGCTCAGAAGAAATGCCATCTAGTGATGATGAGGCTACTGCTGACT
CTCAACATTCTACTCCTCCAAAAAAGAAGAGAAAGGTAGAAGACCCCAAGG
ACTTTCCTTCAGAATTGCTAAGTTTTTTGAGTCATGCTGTGTTTAGTAATAGA
ACTCTTGCTTGCTTTGCTATTTACACCACAAAGGAAAAAGCTGCACTGCTATA
CAAGAAAATTATGGAAAAATATTCTGTAACCTTTATAAGTAGGCATAACAGTT
ATAATCATAACATACTGTTTTTTCTTACTCCACACAGGCATAGAGTGTCTGCTA
TTAATAACTATGCTCAAAAATTGTGTACCTTTAGCTTTTTAATTTGTAAAGGGG
TTAATAAGGAATATTTGATGTATAGTGCCTTGACTAGAGATCCATTTTCTGTTA
TTGAGGAAAGTTTGCCAGGTGGGTTAAAGGAGCATGATTTTAATCCAGAAGA
AGCAGAGGAAACTAAACAAGTGTCCTGGAAGCTTGTAACAGAGTATGCAAT
GGAAACAAAATGTGATGATGTGTTGTTATTGCTTGGGATGTACTTGGAATTTC
AATACAGTTTTGAAATGTGTTTAAAATGTATTAAAAAAGAACAGCCCAGCCA
CTATAAGTACCATGAAAAGCATTATGCAAATGCTGCTATATTTGCTGACAGCA
AAAACCAAAAAACCATATGCCAACAGGCTGTTGATACTGTTTTAGCTAAAAA
GCGGGTTGATAGCCTACAACTAACTAGAGAACAAATGTTAACAAACAGATTT
AATGATCTTTTGGATAGGATGGATATAATGTTTGGTTCTACAGGCTCTGCTGA
CATAGAAGAATGGATGGCTGGAGTTGCTTGGCTACACTGTTTGTTGCCCAAA
ATGGATTCAGTGGTGTATGACTTTTTAAAATGCATGGTGTACAACATTCCTAA
AAAAAGATACTGGCTGTTTAAAGGACCAATTGATAGTGGTAAAACTACATTA
GCAGCTGCTTTGCTTGAATTATGTGGGGGGAAAGCTTTAAATGTTAATTTGCC
CTTGGACAGGCTGAACTTTGAGCTAGGAGTAGCTATTGACCAGTTTTTAGTA
GTTTTTGAGGATGTAAAGGGCACTGGAGGGGAGTCCAGAGATTTGCCTTCA
GGTCAGGGAATTAATAACCTGGACAATTTAAGGGATTATTTGGATGGCAGTGT
TAAGGTAAACTTAGAAAAGAAACACCTAAATAAAAGAACTCAAATATTTCCC
CCTGGAATAGTCACCATGAATGAGTACAGTGTGCCTAAAACACTGCAGGCCA
GATTTGTAAAACAAATAGATTTAGGCCCAAAGATTATTTAAAGCATTGCCTG
GAACGCAGTGAGTTTTTGTTAGAAAAGAGAATAATTCAAAGTGGCATTGCTT

TGCTTCTTATGTTAATTTGGTACAGACCTGTGGCTGAGTTTGCTCAAAGTATT
CAGAGCAGAATTGTGGAGTGGAAAGAGAGATTGGACAAAGAGTTTAGTTTG
TCAGTGTATCAAAAAATGAAGTTTAATGTGGCTATGGGAATTGGAGTTTTAGA
TTGGCTAAGAAACAGTGATGATGATGATGAAGACAGCCAGGAAAATGCTGA
TAAAAATGAAGATGGTGGGGAGAAGAACATGGAAGACTCAGGGCATGAAA
CAGGCATTGATTCACAGTCCCAAGGCTCATTTCAGGCCCCTCAGTCCTCACA
GTCTGTTCATGATCATAATCAGCCATACCACATTTGTAGAGGTTTTACTTGCTT
TAAAAAACCTCCCACACCTCCCCCTGAACCTGAAACAGGATCC
