# Supplementary material for: Establishment and Characterization of an Immortalized Porcine Satellite Cell Line from China Junmu No.1 Pigs
Source: Vet Sci. 2026 Jun 4;13(6):556. doi: 10.3390/vetsci13060556 (PMC13308346; doi:10.3390/vetsci13060556)
Supplement: Supplementary file 1 [file vetsci-13-00556-s001.zip › Supplementary File S2_ Antibody list.pdf]

Supplementary File S2. Antibody list

number	name	Proteintech	Cat No.	Dilution ratio
1	PAX7 Polyclonal antibody	Proteintech	20570-1-AP	1:200
2	MYOD1 Polyclonal antibody	Proteintech	18943-1-AP	1:200
3	anti-SV40 T antigen antibody	MedChemExpress	HY-P83511	1:1000
4	Desmin Polyclonal antibody	Proteintech	16520-1-AP	1:200
5	MYH6 Polyclonal antibody (MYHC)	Proteintech	22281-1-AP	1:200
6	MYOD1 Polyclonal antibody	Proteintech	18943-1-AP	1:200
7	Dystrophin Recombinant antibody	Proteintech	83609-5-RR	1:100
8	Rabbit IgG control Polyclonal antibody	Proteintech	30000-0-AP	1:1000
9	HRP-conjugated Goat Anti-Rabbit IgG(H+L)	Proteintech	SA00001-2	1:20000
10	Multi-rAb™ CoraLite® Plus 488-Goat Anti-Mouse Recombinant Secondary Antibody (H+L)	Proteintech	RGAM002	1:1000
11	Multi-rAb™ CoraLite® Plus 488-Goat Anti-Rabbit Recombinant Secondary Antibody (H+L)	Proteintech	RGAR002	1:1000
12	Multi-rAb™ CoraLite® Plus 594-Goat Anti-Rabbit Recombinant Secondary Antibody (H+L)	Proteintech	RGAR004	1:1000
13	Multi-rAb™ CoraLite® Plus 594-Goat Anti-Mouse Recombinant Secondary Antibody (H+L)	Proteintech	RGAM004	1:1000
