# Supplementary material for: Establishment and Characterization of an Immortalized Porcine Satellite Cell Line from China Junmu No.1 Pigs
Source: Vet Sci. 2026 Jun 4;13(6):556. doi: 10.3390/vetsci13060556 (PMC13308346; doi:10.3390/vetsci13060556)
Supplement: Supplementary file 1 [file vetsci-13-00556-s001.zip › Supplementary File S3_muscle_select_GO_terms.pdf]

Supplementary File S3: Muscle development-related gene set compiled from GO terms

GO:0062086regulation of vein smooth muscle contraction

GO:0062087positive regulation of vein smooth muscle contraction

GO:0062088negative regulation of vein smooth muscle contraction

GO:0062224positive regulation of somatic muscle development

GO:0062225negative regulation of somatic muscle development

GO:0062226regulation of adult somatic muscle development

GO:0062227positive regulation of adult somatic muscle development

GO:0062223regulation of somatic muscle development

GO:0062230negative regulation of larval somatic muscle development

GO:0062231positive regulation of larval somatic muscle development

GO:0062228negative regulation of adult somatic muscle development

GO:0062229regulation of larval somatic muscle development

GO:0120063stomach smooth muscle contraction

GO:0120064stomach pylorus smooth muscle contraction

GO:0120065pyloric antrum smooth muscle contraction

GO:0120066pyloric canal smooth muscle contraction

GO:0120067pyloric sphincter smooth muscle contraction

GO:0120068regulation of stomach fundus smooth muscle contraction

GO:0120069positive regulation of stomach fundus smooth muscle contraction

GO:0120072positive regulation of pyloric antrum smooth muscle contraction

GO:0120073negative regulation of pyloric antrum smooth muscle contraction

GO:0120070negative regulation of stomach fundus smooth muscle contraction

GO:0120071regulation of pyloric antrum smooth muscle contraction

GO:0099622cardiac muscle cell membrane repolarization

GO:0099623regulation of cardiac muscle cell membrane repolarization

GO:0099624atrial cardiac muscle cell membrane repolarization

GO:0099625ventricular cardiac muscle cell membrane repolarization

GO:1904320positive regulation of smooth muscle contraction involved in micturition

GO:1904304regulation of gastro-intestinal system smooth muscle contraction

GO:1904305negative regulation of gastro-intestinal system smooth muscle contraction

GO:1904306positive regulation of gastro-intestinal system smooth muscle contraction

GO:1904318regulation of smooth muscle contraction involved in micturition

GO:1904319negative regulation of smooth muscle contraction involved in micturition

GO:1904343positive regulation of colon smooth muscle contraction

GO:1904341regulation of colon smooth muscle contraction

GO:1904342negative regulation of colon smooth muscle contraction

GO:1904347regulation of small intestine smooth muscle contraction

GO:1904348negative regulation of small intestine smooth muscle contraction

GO:1904349positive regulation of small intestine smooth muscle contraction

GO:1904394negative regulation of skeletal muscle acetylcholine-gated channel clustering

GO:1904395positive regulation of skeletal muscle acetylcholine-gated channel clustering

GO:1904393regulation of skeletal muscle acetylcholine-gated channel clustering

GO:1903243negative regulation of cardiac muscle hypertrophy in response to stress

GO:1903244positive regulation of cardiac muscle hypertrophy in response to stress

GO:1903242regulation of cardiac muscle hypertrophy in response to stress

GO:1903760regulation of voltage-gated potassium channel activity involved in ventricular cardiac muscle cell action potential repolarization

GO:0110021cardiac muscle myoblast proliferation

GO:0110024positive regulation of cardiac muscle myoblast proliferation

GO:0110022regulation of cardiac muscle myoblast proliferation

GO:0110023negative regulation of cardiac muscle myoblast proliferation

GO:1904204regulation of skeletal muscle hypertrophy

GO:1904205negative regulation of skeletal muscle hypertrophy

GO:1904206positive regulation of skeletal muscle hypertrophy

GO:1904113negative regulation of muscle filament sliding

GO:1904114positive regulation of muscle filament sliding

GO:1904198negative regulation of regulation of vascular associated smooth muscle cell membrane depolarization

GO:1904199positive regulation of regulation of vascular associated smooth muscle cell membrane depolarization

GO:1904969slow muscle cell migration

GO:1905609positive regulation of smooth muscle cell-matrix adhesion

GO:1905656positive regulation of artery smooth muscle contraction

GO:1905655negative regulation of artery smooth muscle contraction

GO:1905654regulation of artery smooth muscle contraction

GO:1904695positive regulation of vascular associated smooth muscle contraction

GO:1904694negative regulation of vascular associated smooth muscle contraction

GO:1904738vascular associated smooth muscle cell migration

GO:1904707positive regulation of vascular associated smooth muscle cell proliferation

GO:1904706negative regulation of vascular associated smooth muscle cell proliferation

GO:1904705regulation of vascular associated smooth muscle cell proliferation

GO:1904754positive regulation of vascular associated smooth muscle cell migration

GO:1904753negative regulation of vascular associated smooth muscle cell migration

GO:1904752regulation of vascular associated smooth muscle cell migration

GO:1904829regulation of aortic smooth muscle cell differentiation

GO:1904831positive regulation of aortic smooth muscle cell differentiation

GO:1904830negative regulation of aortic smooth muscle cell differentiation

GO:1905026positive regulation of membrane repolarization during ventricular cardiac muscle cell action potential

GO:1905025negative regulation of membrane repolarization during ventricular cardiac muscle cell action potential

GO:1905024regulation of membrane repolarization during ventricular cardiac muscle cell action potential

GO:1905000regulation of membrane repolarization during atrial cardiac muscle cell action potential

GO:1905002positive regulation of membrane repolarization during atrial cardiac muscle cell action potential

GO:1905001negative regulation of membrane repolarization during atrial cardiac muscle cell action potential

GO:1905065positive regulation of vascular associated smooth muscle cell differentiation

GO:1905064negative regulation of vascular associated smooth muscle cell differentiation

GO:1905063regulation of vascular associated smooth muscle cell differentiation

GO:1905033positive regulation of membrane repolarization during cardiac muscle cell action potential

GO:1905032negative regulation of membrane repolarization during cardiac muscle cell action potential

GO:1905031regulation of membrane repolarization during cardiac muscle cell action potential

GO:1905147regulation of smooth muscle hypertrophy

GO:1905149positive regulation of smooth muscle hypertrophy

GO:1905148negative regulation of smooth muscle hypertrophy

GO:1905176positive regulation of vascular associated smooth muscle cell dedifferentiation

GO:1905175negative regulation of vascular associated smooth muscle cell dedifferentiation

GO:1905174regulation of vascular associated smooth muscle cell dedifferentiation

GO:1905179negative regulation of cardiac muscle tissue regeneration

GO:1905178regulation of cardiac muscle tissue regeneration

GO:1905180positive regulation of cardiac muscle tissue regeneration

GO:1905420vascular associated smooth muscle cell differentiation involved in phenotypic switching

GO:1905459regulation of vascular associated smooth muscle cell apoptotic process

GO:1905461positive regulation of vascular associated smooth muscle cell apoptotic process

GO:1905460negative regulation of vascular associated smooth muscle cell apoptotic process

GO:0060038cardiac muscle cell proliferation

GO:0060045positive regulation of cardiac muscle cell proliferation

GO:0060044negative regulation of cardiac muscle cell proliferation

GO:0060043regulation of cardiac muscle cell proliferation

GO:0060048cardiac muscle contraction

GO:0060085smooth muscle relaxation of the bladder outlet

GO:0060083smooth muscle contraction involved in micturition

GO:0060087relaxation of vascular associated smooth muscle

GO:1905288vascular associated smooth muscle cell apoptotic process

GO:0060307regulation of ventricular cardiac muscle cell membrane repolarization

GO:0060371regulation of atrial cardiac muscle cell membrane depolarization

GO:0060373regulation of ventricular cardiac muscle cell membrane depolarization

GO:0060372regulation of atrial cardiac muscle cell membrane repolarization

GO:0060379cardiac muscle cell myoblast differentiation

GO:0060530smooth muscle cell differentiation involved in prostate glandular acinus development

GO:0060537muscle tissue development

GO:0060538skeletal muscle organ development

GO:0060415muscle tissue morphogenesis

GO:0060414aorta smooth muscle tissue morphogenesis

GO:0060452positive regulation of cardiac muscle contraction

GO:0035914skeletal muscle cell differentiation

GO:0061026cardiac muscle tissue regeneration

GO:0061203striated muscle paramyosin thick filament assembly

GO:0061214mesonephric smooth muscle tissue development

GO:0061199striated muscle contraction involved in embryonic body morphogenesis

GO:0036194muscle cell projection

GO:0036195muscle cell projection membrane

GO:0061145lung smooth muscle development

GO:0061061muscle structure development

GO:0036061muscle cell chemotaxis toward tendon cell

GO:0061049cell growth involved in cardiac muscle cell development

GO:0061050regulation of cell growth involved in cardiac muscle cell development

GO:0061051positive regulation of cell growth involved in cardiac muscle cell development

GO:0061052negative regulation of cell growth involved in cardiac muscle cell development

GO:0072208metanephric smooth muscle tissue development

GO:0072191ureter smooth muscle development

GO:0072195kidney smooth muscle cell differentiation

GO:0072193ureter smooth muscle cell differentiation

GO:0072194kidney smooth muscle tissue development

GO:1902282voltage-gated potassium channel activity involved in ventricular cardiac muscle cell action potential repolarization

GO:2000098negative regulation of smooth muscle cell-matrix adhesion

GO:2000097regulation of smooth muscle cell-matrix adhesion

GO:2000063positive regulation of ureter smooth muscle cell differentiation

GO:2000062negative regulation of ureter smooth muscle cell differentiation

GO:2000061regulation of ureter smooth muscle cell differentiation

GO:0048641regulation of skeletal muscle tissue development

GO:0048644muscle organ morphogenesis

GO:0048643positive regulation of skeletal muscle tissue development

GO:0048642negative regulation of skeletal muscle tissue development

GO:0048636positive regulation of muscle organ development

GO:0048635negative regulation of muscle organ development

GO:0048630skeletal muscle tissue growth

GO:0048634regulation of muscle organ development

GO:0048633positive regulation of skeletal muscle tissue growth

GO:0048632negative regulation of skeletal muscle tissue growth

GO:0048631regulation of skeletal muscle tissue growth

GO:0034392negative regulation of smooth muscle cell apoptotic process

GO:0034391regulation of smooth muscle cell apoptotic process

GO:0034390smooth muscle cell apoptotic process

GO:0034393positive regulation of smooth muscle cell apoptotic process

GO:0061302smooth muscle cell-matrix adhesion

GO:0061345planar cell polarity pathway involved in cardiac muscle cell fate commitment

GO:0061442cardiac muscle cell fate determination

GO:0060948cardiac vascular smooth muscle cell development

GO:0060947cardiac vascular smooth muscle cell differentiation

GO:0060949cardiac vascular smooth muscle cell fate commitment

GO:0035995detection of muscle stretch

GO:0035994response to muscle stretch

GO:0035981tongue muscle cell differentiation

GO:0060925ventricular cardiac muscle cell fate commitment

GO:0060924atrial cardiac muscle cell fate commitment

GO:0060923cardiac muscle cell fate commitment

GO:0060985epicardium-derived cardiac vascular smooth muscle cell fate commitment

GO:0060984epicardium-derived cardiac vascular smooth muscle cell development

GO:0060983epicardium-derived cardiac vascular smooth muscle cell differentiation

GO:0035887aortic smooth muscle cell differentiation

GO:0035886vascular associated smooth muscle cell differentiation

GO:0035645enteric smooth muscle cell differentiation

GO:0100001regulation of skeletal muscle contraction by action potential

GO:2001036negative regulation of tongue muscle cell differentiation

GO:2001037positive regulation of tongue muscle cell differentiation

GO:2001035regulation of tongue muscle cell differentiation

GO:2001014regulation of skeletal muscle cell differentiation

GO:2001015negative regulation of skeletal muscle cell differentiation

GO:2001016positive regulation of skeletal muscle cell differentiation

GO:2001281regulation of muscle cell chemotaxis toward tendon cell

GO:2001282negative regulation of muscle cell chemotaxis toward tendon cell

GO:2001283Roundabout signaling pathway involved in muscle cell chemotaxis toward tendon cell

GO:0140137venom-mediated perturbation of muscle system process

GO:0010882regulation of cardiac muscle contraction by calcium ion signaling

GO:0010881regulation of cardiac muscle contraction by regulation of the release of sequestered calcium ion

GO:0006936muscle contraction

GO:0006937regulation of muscle contraction

GO:0006939smooth muscle contraction

GO:0006940regulation of smooth muscle contraction

GO:1903948negative regulation of atrial cardiac muscle cell action potential

GO:1903949positive regulation of atrial cardiac muscle cell action potential

GO:0006941striated muscle contraction

GO:1903946negative regulation of ventricular cardiac muscle cell action potential

GO:0006942regulation of striated muscle contraction

GO:1903947positive regulation of ventricular cardiac muscle cell action potential

GO:2000357negative regulation of kidney smooth muscle cell differentiation

GO:2000356regulation of kidney smooth muscle cell differentiation

GO:2000358positive regulation of kidney smooth muscle cell differentiation

GO:2000727positive regulation of cardiac muscle cell differentiation

GO:2000726negative regulation of cardiac muscle cell differentiation

GO:2000725regulation of cardiac muscle cell differentiation

GO:2000724positive regulation of cardiac vascular smooth muscle cell differentiation

GO:2000723negative regulation of cardiac vascular smooth muscle cell differentiation

GO:2000722regulation of cardiac vascular smooth muscle cell differentiation

GO:2000700positive regulation of cardiac muscle cell myoblast differentiation

GO:0086022SA node cell-atrial cardiac muscle cell adhesion involved in cell communication

GO:0086020gap junction channel activity involved in SA node cell-atrial cardiac muscle cell electrical coupling

GO:0086021SA node cell to atrial cardiac muscle cell communication by electrical coupling

GO:0086026atrial cardiac muscle cell to AV node cell signaling

GO:0086029Purkinje myocyte to ventricular cardiac muscle cell signaling

GO:0086012membrane depolarization during cardiac muscle cell action potential

GO:0086013membrane repolarization during cardiac muscle cell action potential

GO:0086014atrial cardiac muscle cell action potential

GO:0086018SA node cell to atrial cardiac muscle cell signaling

GO:0086001cardiac muscle cell action potential

GO:0086004regulation of cardiac muscle cell contraction

GO:0086005ventricular cardiac muscle cell action potential

GO:0086002cardiac muscle cell action potential involved in contraction

GO:0086003cardiac muscle cell contraction

GO:0086008voltage-gated potassium channel activity involved in cardiac muscle cell action potential repolarization

GO:0086006voltage-gated sodium channel activity involved in cardiac muscle cell action potential

GO:0086007voltage-gated calcium channel activity involved in cardiac muscle cell action potential

GO:0086066atrial cardiac muscle cell to AV node cell communication

GO:0086068Purkinje myocyte to ventricular cardiac muscle cell communication

GO:0086055Purkinje myocyte to ventricular cardiac muscle cell communication by electrical coupling

GO:0086040sodium:proton antiporter activity involved in regulation of cardiac muscle cell membrane potential

GO:0086044atrial cardiac muscle cell to AV node cell communication by electrical coupling

GO:0086042cardiac muscle cell-cardiac muscle cell adhesion

GO:0086030adenylate cyclase-activating adrenergic receptor signaling pathway involved in cardiac muscle relaxation

GO:0086037P-type sodium:potassium-exchanging transporter activity involved in regulation of cardiac muscle cell membrane potential

GO:0086038calcium:sodium antiporter activity involved in regulation of cardiac

muscle cell membrane potential

GO:0086036regulation of cardiac muscle cell membrane potential

GO:0086039P-type calcium transporter activity involved in regulation of cardiac muscle cell membrane potential

GO:0086084cell adhesive protein binding involved in Purkinje myocyte-ventricular cardiac muscle cell communication

GO:0086085cell adhesive protein binding involved in SA cardiac muscle cell-atrial cardiac muscle cell communication

GO:0086089voltage-gated potassium channel activity involved in atrial cardiac muscle cell action potential repolarization

GO:1905899regulation of smooth muscle tissue development

GO:1905901positive regulation of smooth muscle tissue development

GO:1905900negative regulation of smooth muscle tissue development

GO:1905932positive regulation of vascular associated smooth muscle cell differentiation involved in phenotypic switching

GO:1905931negative regulation of vascular associated smooth muscle cell differentiation involved in phenotypic switching

GO:1905930regulation of vascular associated smooth muscle cell differentiation involved in phenotypic switching

GO:2000690regulation of cardiac muscle cell myoblast differentiation

GO:2000691negative regulation of cardiac muscle cell myoblast differentiation

GO:0086081cell adhesive protein binding involved in atrial cardiac muscle cell-AV node cell communication

GO:0086074Purkinje myocyte-ventricular cardiac muscle cell adhesion involved in cell communication

GO:0086071atrial cardiac muscle cell-AV node cell adhesion involved in cell communication

GO:0086076gap junction channel activity involved in atrial cardiac muscle cell-AV node cell electrical coupling

GO:0086079gap junction channel activity involved in Purkinje myocyte-ventricular

cardiac muscle cell electrical coupling

GO:0086070SA node cell to atrial cardiac muscle cell communication

GO:0048745smooth muscle tissue development

GO:0048744negative regulation of skeletal muscle fiber development

GO:0048743positive regulation of skeletal muscle fiber development

GO:0048742regulation of skeletal muscle fiber development

GO:0048741skeletal muscle fiber development

GO:0048738cardiac muscle tissue development

GO:0048662negative regulation of smooth muscle cell proliferation

GO:0048661positive regulation of smooth muscle cell proliferation

GO:0048660regulation of smooth muscle cell proliferation

GO:0048659smooth muscle cell proliferation

GO:0010613positive regulation of cardiac muscle hypertrophy

GO:0010612regulation of cardiac muscle adaptation

GO:0010615positive regulation of cardiac muscle adaptation

GO:0010614negative regulation of cardiac muscle hypertrophy

GO:0010611regulation of cardiac muscle hypertrophy

GO:0010616negative regulation of cardiac muscle adaptation

GO:0010657muscle cell apoptotic process

GO:0010656negative regulation of muscle cell apoptotic process

GO:0010659cardiac muscle cell apoptotic process

GO:0010658striated muscle cell apoptotic process

GO:0010667negative regulation of cardiac muscle cell apoptotic process

GO:0010664negative regulation of striated muscle cell apoptotic process

GO:0010663positive regulation of striated muscle cell apoptotic process

GO:0010666positive regulation of cardiac muscle cell apoptotic process

GO:0010665regulation of cardiac muscle cell apoptotic process

GO:0010660regulation of muscle cell apoptotic process

GO:0010662regulation of striated muscle cell apoptotic process

GO:0010661positive regulation of muscle cell apoptotic process

GO:0090078smooth muscle derived foam cell differentiation

GO:0090076relaxation of skeletal muscle

GO:0090075relaxation of muscle

GO:0090257regulation of muscle system process

GO:0051371muscle alpha-actinin binding

GO:0051149positive regulation of muscle cell differentiation

GO:0051147regulation of muscle cell differentiation

GO:0051148negative regulation of muscle cell differentiation

GO:0051145smooth muscle cell differentiation

GO:0051146striated muscle cell differentiation

GO:0051152positive regulation of smooth muscle cell differentiation

GO:0051153regulation of striated muscle cell differentiation

GO:0051150regulation of smooth muscle cell differentiation

GO:0051151negative regulation of smooth muscle cell differentiation

GO:0051154negative regulation of striated muscle cell differentiation

GO:0051155positive regulation of striated muscle cell differentiation

GO:0106135negative regulation of cardiac muscle cell contraction

GO:0106134positive regulation of cardiac muscle cell contraction

GO:0032971regulation of muscle filament sliding

GO:0032972regulation of muscle filament sliding speed

GO:1902723negative regulation of skeletal muscle satellite cell proliferation

GO:1902724positive regulation of skeletal muscle satellite cell proliferation

GO:1902727negative regulation of growth factor dependent skeletal muscle satellite cell proliferation

GO:1902728positive regulation of growth factor dependent skeletal muscle satellite cell proliferation

GO:1902726positive regulation of skeletal muscle satellite cell differentiation

GO:1902766skeletal muscle satellite cell migration

GO:0007519skeletal muscle tissue development

GO:0007517muscle organ development

GO:0007522visceral muscle development

GO:0007523larval visceral muscle development

GO:0007521muscle cell fate determination

GO:0007526larval somatic muscle development

GO:0007527adult somatic muscle development

GO:0007524adult visceral muscle development

GO:0007525somatic muscle development

GO:1902809regulation of skeletal muscle fiber differentiation

GO:1902811positive regulation of skeletal muscle fiber differentiation

GO:1902810negative regulation of skeletal muscle fiber differentiation

GO:0033002muscle cell proliferation

GO:0005927muscle tendon junction

GO:0030240skeletal muscle thin filament assembly

GO:0030241skeletal muscle myosin thick filament assembly

GO:0030486smooth muscle dense body

GO:0030485smooth muscle contractile fiber

GO:0005859muscle myosin complex

GO:0005862muscle thin filament tropomyosin

GO:0005863striated muscle myosin thick filament

GO:0005865striated muscle thin filament

GO:0044617venom-mediated smooth muscle relaxation

GO:0030049muscle filament sliding

GO:0003009skeletal muscle contraction

GO:0003010voluntary skeletal muscle contraction

GO:0003011involuntary skeletal muscle contraction

GO:0003012muscle system process

GO:0003056regulation of vascular associated smooth muscle contraction

GO:1990874vascular associated smooth muscle cell proliferation

GO:0003220left ventricular cardiac muscle tissue morphogenesis

GO:0003221right ventricular cardiac muscle tissue morphogenesis

GO:0003228atrial cardiac muscle tissue development

GO:0003229ventricular cardiac muscle tissue development

GO:0003245cardiac muscle tissue growth involved in heart morphogenesis

GO:0003246embryonic cardiac muscle cell growth involved in heart morphogenesis

GO:0003247post-embryonic cardiac muscle cell growth involved in heart morphogenesis

GO:0003261cardiac muscle progenitor cell migration to the midline involved in heart field formation

GO:0003298physiological muscle hypertrophy

GO:0003299muscle hypertrophy in response to stress

GO:1990936vascular associated smooth muscle cell dedifferentiation

GO:0042692muscle cell differentiation

GO:0042693muscle cell fate commitment

GO:0042694muscle cell fate specification

GO:0160096nematode pharyngeal muscle development

GO:0160175somatic muscle attachment to chitin-based cuticle

GO:0097081vascular associated smooth muscle cell fate commitment

GO:0097083vascular associated smooth muscle cell fate determination

GO:0097082vascular associated smooth muscle cell fate specification

GO:0097084vascular associated smooth muscle cell development

GO:0097482muscle cell postsynaptic specialization

GO:0097365stretch-activated, monoatomic cation-selective, calcium channel activity involved in regulation of cardiac muscle cell action potential

GO:0098522neuromuscular junction of skeletal muscle fiber

GO:0098524neuromuscular junction of somatic muscle myotube

GO:0098527neuromuscular junction of somatic muscle

GO:0098723skeletal muscle myofibril

GO:0098726symmetric division of skeletal muscle satellite stem cell

GO:0098731skeletal muscle satellite stem cell maintenance involved in skeletal muscle regeneration

GO:0098529neuromuscular junction development, skeletal muscle fiber

GO:0098528skeletal muscle fiber differentiation

GO:0070471uterine smooth muscle contraction

GO:0070473negative regulation of uterine smooth muscle contraction

GO:0070472regulation of uterine smooth muscle contraction

GO:0070474positive regulation of uterine smooth muscle contraction

GO:0098901regulation of cardiac muscle cell action potential

GO:0098909regulation of cardiac muscle cell action potential involved in regulation of contraction

GO:0098910regulation of atrial cardiac muscle cell action potential

GO:0098912membrane depolarization during atrial cardiac muscle cell action potential

GO:0098911regulation of ventricular cardiac muscle cell action potential

GO:0098914membrane repolarization during atrial cardiac muscle cell action potential

GO:0098913membrane depolarization during ventricular cardiac muscle cell action potential

GO:0098915membrane repolarization during ventricular cardiac muscle cell action potential

GO:0055120striated muscle dense body

GO:0055117regulation of cardiac muscle contraction

GO:0055118negative regulation of cardiac muscle contraction

GO:0055119relaxation of cardiac muscle

GO:0055007cardiac muscle cell differentiation

GO:0055008cardiac muscle tissue morphogenesis

GO:0055009atrial cardiac muscle tissue morphogenesis

GO:0055001muscle cell development

GO:0055002striated muscle cell development

GO:0055015ventricular cardiac muscle cell development

GO:0055017cardiac muscle tissue growth

GO:0055018regulation of cardiac muscle fiber development

GO:0055019negative regulation of cardiac muscle fiber development

GO:0055010ventricular cardiac muscle tissue morphogenesis

GO:0055011atrial cardiac muscle cell differentiation

GO:0055012ventricular cardiac muscle cell differentiation

GO:0055013cardiac muscle cell development

GO:0055014atrial cardiac muscle cell development

GO:0055026negative regulation of cardiac muscle tissue development

GO:0055020positive regulation of cardiac muscle fiber development

GO:0055021regulation of cardiac muscle tissue growth

GO:0055022negative regulation of cardiac muscle tissue growth

GO:0055023positive regulation of cardiac muscle tissue growth

GO:0055024regulation of cardiac muscle tissue development

GO:0055025positive regulation of cardiac muscle tissue development

GO:0002074extraocular skeletal muscle development

GO:0002075somitomeric trunk muscle development

GO:0016202regulation of striated muscle tissue development

GO:0016203muscle attachment

GO:0016204determination of muscle attachment site

GO:0043501skeletal muscle adaptation

GO:0043500muscle adaptation

GO:0043503skeletal muscle fiber adaptation

GO:0043502regulation of muscle adaptation

GO:0043415positive regulation of skeletal muscle tissue regeneration

GO:0043417negative regulation of skeletal muscle tissue regeneration

GO:0043416regulation of skeletal muscle tissue regeneration

GO:0043403skeletal muscle tissue regeneration

GO:1901077regulation of relaxation of muscle

GO:1901079positive regulation of relaxation of muscle

GO:1901078negative regulation of relaxation of muscle

GO:1901080regulation of relaxation of smooth muscle

GO:1901082positive regulation of relaxation of smooth muscle

GO:1901081negative regulation of relaxation of smooth muscle

GO:1901897regulation of relaxation of cardiac muscle

GO:1901899positive regulation of relaxation of cardiac muscle

GO:1901898negative regulation of relaxation of cardiac muscle

GO:0043292contractile muscle fiber

GO:0043282chordate pharyngeal muscle development

GO:1901862negative regulation of muscle tissue development

GO:1901861regulation of muscle tissue development

GO:1901863positive regulation of muscle tissue development

GO:1901667negative regulation of skeletal muscle satellite cell activation involved in skeletal muscle regeneration

GO:0045986negative regulation of smooth muscle contraction

GO:0045987positive regulation of smooth muscle contraction

GO:0045988negative regulation of striated muscle contraction

GO:0045989positive regulation of striated muscle contraction

GO:0045932negative regulation of muscle contraction

GO:0045933positive regulation of muscle contraction

GO:0031451positive regulation of slow-twitch skeletal muscle fiber contraction

GO:0031450negative regulation of slow-twitch skeletal muscle fiber contraction

GO:0031444slow-twitch skeletal muscle fiber contraction

GO:0031443fast-twitch skeletal muscle fiber contraction

GO:0031448positive regulation of fast-twitch skeletal muscle fiber contraction

GO:0031449regulation of slow-twitch skeletal muscle fiber contraction

GO:0031446regulation of fast-twitch skeletal muscle fiber contraction

GO:0031447negative regulation of fast-twitch skeletal muscle fiber contraction

GO:0045843negative regulation of striated muscle tissue development

GO:0045844positive regulation of striated muscle tissue development

GO:0008307structural constituent of muscle

GO:1900720negative regulation of uterine smooth muscle relaxation

GO:1900721positive regulation of uterine smooth muscle relaxation

GO:1900719regulation of uterine smooth muscle relaxation

GO:1900825regulation of membrane depolarization during cardiac muscle cell action potential

GO:1900827positive regulation of membrane depolarization during cardiac muscle cell action potential

GO:1900826negative regulation of membrane depolarization during cardiac muscle cell action potential

GO:0071688striated muscle myosin thick filament assembly

GO:0071689muscle thin filament assembly

GO:0071690cardiac muscle myosin thick filament assembly

GO:0071691cardiac muscle thin filament assembly

GO:0071671regulation of smooth muscle cell chemotaxis

GO:0071672negative regulation of smooth muscle cell chemotaxis

GO:0071670smooth muscle cell chemotaxis

GO:0071673positive regulation of smooth muscle cell chemotaxis

GO:0044521venom-mediated muscle damage in another organism

GO:0044557relaxation of smooth muscle

GO:0044558uterine smooth muscle relaxation

GO:0071340skeletal muscle acetylcholine-gated channel clustering

GO:0046716muscle cell cellular homeostasis

GO:1990736regulation of vascular associated smooth muscle cell membrane depolarization

GO:1990765colon smooth muscle contraction

GO:1990770small intestine smooth muscle contraction

GO:0003300cardiac muscle hypertrophy

GO:0003301physiological cardiac muscle hypertrophy

GO:0014905myoblast fusion involved in skeletal muscle regeneration

GO:0014901satellite cell activation involved in skeletal muscle regeneration

GO:0014900muscle hyperplasia

GO:0014909smooth muscle cell migration

GO:0014908myotube differentiation involved in skeletal muscle regeneration

GO:0014906myotube cell development involved in skeletal muscle regeneration

GO:0014915regulation of muscle filament sliding speed involved in regulation of the velocity of shortening in skeletal muscle contraction

GO:0014914myoblast maturation involved in muscle regeneration

GO:0014912negative regulation of smooth muscle cell migration

GO:0014911positive regulation of smooth muscle cell migration

GO:0014910regulation of smooth muscle cell migration

GO:0014899cardiac muscle atrophy

GO:0014898cardiac muscle hypertrophy in response to stress

GO:0014897striated muscle hypertrophy

GO:0014896muscle hypertrophy

GO:0014895smooth muscle hypertrophy

GO:0014894response to denervation involved in regulation of muscle adaptation

GO:0014893response to rest involved in regulation of muscle adaptation

GO:0014891striated muscle atrophy

GO:0014890smooth muscle atrophy

GO:0014709positive regulation of somitomeric trunk muscle development

GO:0014708regulation of somitomeric trunk muscle development

GO:0014718positive regulation of satellite cell activation involved in skeletal muscle regeneration

GO:0014717regulation of satellite cell activation involved in skeletal muscle regeneration

GO:0014716skeletal muscle satellite stem cell asymmetric division involved in skeletal muscle regeneration

GO:0014713negative regulation of branchiomeric skeletal muscle development

GO:0014712positive regulation of branchiomeric skeletal muscle development

GO:0014711regulation of branchiomeric skeletal muscle development

GO:0014710negative regulation of somitomeric trunk muscle development

GO:0014719skeletal muscle satellite cell activation

GO:0014729regulation of the velocity of shortening of skeletal muscle modulating contraction

GO:0014728regulation of the force of skeletal muscle contraction

GO:0014727positive regulation of extraocular skeletal muscle development

GO:0014726negative regulation of extraocular skeletal muscle development

GO:0014725regulation of extraocular skeletal muscle development

GO:0014724regulation of twitch skeletal muscle contraction

GO:0014723regulation of skeletal muscle contraction by modulation of calcium ion sensitivity of myofibril

GO:0014722regulation of skeletal muscle contraction by calcium ion signaling

GO:0014721twitch skeletal muscle contraction

GO:0014720tonic skeletal muscle contraction

GO:0014739positive regulation of muscle hyperplasia

GO:0014738regulation of muscle hyperplasia

GO:0014737positive regulation of muscle atrophy

GO:0014736negative regulation of muscle atrophy

GO:0014735regulation of muscle atrophy

GO:0014734skeletal muscle hypertrophy

GO:0014733regulation of skeletal muscle adaptation

GO:0014732skeletal muscle atrophy

GO:0014730skeletal muscle regeneration at neuromuscular junction

GO:0014748negative regulation of tonic skeletal muscle contraction

GO:0014747positive regulation of tonic skeletal muscle contraction

GO:0014746regulation of tonic skeletal muscle contraction

GO:0014745negative regulation of muscle adaptation

GO:0014744positive regulation of muscle adaptation

GO:0014743regulation of muscle hypertrophy

GO:0014742positive regulation of muscle hypertrophy

GO:0014741negative regulation of muscle hypertrophy

GO:0014740negative regulation of muscle hyperplasia

GO:0014806smooth muscle hyperplasia

GO:0014805smooth muscle adaptation

GO:0014809regulation of skeletal muscle contraction by regulation of release of sequestered calcium ion

GO:0014817skeletal muscle satellite cell fate specification

GO:0014816skeletal muscle satellite cell differentiation

GO:0014813skeletal muscle satellite cell commitment

GO:0014812muscle cell migration

GO:0014811negative regulation of skeletal muscle contraction by regulation of release of sequestered calcium ion

GO:0014810positive regulation of skeletal muscle contraction by regulation of release of sequestered calcium ion

GO:0014819regulation of skeletal muscle contraction

GO:0014818skeletal muscle satellite cell fate determination

GO:0014828distal stomach smooth muscle contraction

GO:0014827intestine smooth muscle contraction

GO:0014826vein smooth muscle contraction

GO:0014825stomach fundus smooth muscle contraction

GO:0014824artery smooth muscle contraction

GO:0014821phasic smooth muscle contraction

GO:0014820tonic smooth muscle contraction

GO:0014829vascular associated smooth muscle contraction

GO:0014839myoblast migration involved in skeletal muscle regeneration

GO:0014838myoblast fate specification involved in skeletal muscle regeneration

GO:0014837myoblast fate determination involved in skeletal muscle regeneration

GO:0014836myoblast fate commitment involved in skeletal muscle regeneration

GO:0014835myoblast differentiation involved in skeletal muscle regeneration

GO:0014834skeletal muscle satellite cell maintenance involved in skeletal muscle regeneration

GO:0014833skeletal muscle satellite stem cell asymmetric division

GO:0014832urinary bladder smooth muscle contraction

GO:0014831gastro-intestinal system smooth muscle contraction

GO:0014830arteriole smooth muscle contraction

GO:0014849ureter smooth muscle contraction

GO:0014848urinary tract smooth muscle contraction

GO:0014847proximal stomach smooth muscle contraction

GO:0014846esophagus smooth muscle contraction

GO:0014845stomach body smooth muscle contraction

GO:0014844myoblast proliferation involved in skeletal muscle regeneration

GO:0014843growth factor dependent regulation of skeletal muscle satellite cell proliferation

GO:0014842regulation of skeletal muscle satellite cell proliferation

GO:0014841skeletal muscle satellite cell proliferation

GO:0014859negative regulation of skeletal muscle cell proliferation

GO:0014858positive regulation of skeletal muscle cell proliferation

GO:0014857regulation of skeletal muscle cell proliferation

GO:0014856skeletal muscle cell proliferation

GO:0014855striated muscle cell proliferation

GO:0014853regulation of excitatory postsynaptic membrane potential involved in skeletal muscle contraction

GO:0014852regulation of skeletal muscle contraction by neural stimulation via neuromuscular junction

GO:0014850response to muscle activity

GO:0014869detection of muscle inactivity

GO:0014868cross bridge cycling involved in regulation of the velocity of shortening

in skeletal muscle contraction

GO:0014864detection of muscle activity

GO:0014862regulation of skeletal muscle contraction by chemo-mechanical energy conversion

GO:0014861regulation of skeletal muscle contraction via regulation of action potential

GO:0014860neurotransmitter secretion involved in regulation of skeletal muscle contraction

GO:0014879detection of electrical stimulus involved in regulation of muscle adaptation

GO:0014878response to electrical stimulus involved in regulation of muscle adaptation

GO:0014877response to muscle inactivity involved in regulation of muscle adaptation

GO:0014876response to injury involved in regulation of muscle adaptation

GO:0014875detection of muscle activity involved in regulation of muscle adaptation

GO:0014874response to stimulus involved in regulation of muscle adaptation

GO:0014873response to muscle activity involved in regulation of muscle adaptation

GO:0014871cross bridge formation involved in regulation of the velocity of shortening in skeletal muscle contraction

GO:0014870response to muscle inactivity

GO:0014889muscle atrophy

GO:0014888striated muscle adaptation

GO:0014887cardiac muscle adaptation

GO:0014885detection of injury involved in regulation of muscle adaptation

GO:0014884detection of muscle inactivity involved in regulation of muscle adaptation

GO:0014880regulation of muscle filament sliding involved in regulation of the velocity of shortening in skeletal muscle contraction

GO:0014707branchiomeric skeletal muscle development

GO:0014706striated muscle tissue development

GO:0014703oscillatory muscle contraction

GO:0099153synaptic transmission, serotonergic

GO:0099155synaptic transmission, noradrenergic

GO:0035249synaptic transmission, glutamatergic

GO:0060298positive regulation of sarcomere organization

GO:0060297regulation of sarcomere organization

GO:0060299negative regulation of sarcomere organization

GO:1905029positive regulation of membrane depolarization during AV node cell action potential

GO:1905028negative regulation of membrane depolarization during AV node cell action potential

GO:1905027regulation of membrane depolarization during AV node cell action potential

GO:0060005vestibular reflex

GO:0060004reflex

GO:0060012synaptic transmission, glycinergic

GO:0060007linear vestibuloocular reflex

GO:0060006angular vestibuloocular reflex

GO:0060073micturition

GO:0060084synaptic transmission involved in micturition

GO:0035418protein localization to synapse

GO:0060539diaphragm development

GO:0060451negative regulation of hindgut contraction

GO:0060450positive regulation of hindgut contraction

GO:0036166phenotypic switching

GO:0061174type I terminal bouton

GO:0061176type Ib terminal bouton

GO:0061177type Is terminal bouton

GO:0061073ciliary body morphogenesis

GO:0061055myotome development

GO:0072001renal system development

GO:0007637proboscis extension reflex

GO:0048627myoblast development

GO:0048626myoblast fate specification

GO:0048625myoblast fate commitment

GO:0048628myoblast maturation

GO:0021611facial nerve formation

GO:0021610facial nerve morphogenesis

GO:0021615glossopharyngeal nerve morphogenesis

GO:0021614glossopharyngeal nerve maturation

GO:0021613facial nerve maturation

GO:0021612facial nerve structural organization

GO:0021608accessory nerve formation

GO:0021607accessory nerve morphogenesis

GO:0021606accessory nerve maturation

GO:0021609accessory nerve structural organization

GO:0021523somatic motor neuron differentiation

GO:0021522spinal cord motor neuron differentiation

GO:0021526medial motor column neuron differentiation

GO:0021559trigeminal nerve development

GO:0021558trochlear nerve development

GO:0021557oculomotor nerve development

GO:0021566hypoglossal nerve development

GO:0021565accessory nerve development

GO:0021563glossopharyngeal nerve development

GO:0021561facial nerve development

GO:0036371protein localization to T-tubule

GO:0036379myofilament

GO:0061337cardiac conduction

GO:0140091mBAF complex

GO:0140074cardiac endothelial to mesenchymal transition

GO:0035990tendon cell differentiation

GO:0035993deltoid tuberosity development

GO:0035989tendon development

GO:0060933His-Purkinje system cell development

GO:0060932His-Purkinje system cell differentiation

GO:0060934His-Purkinje system cell fate commitment

GO:0060982coronary artery morphogenesis

GO:0035882defecation rhythm

GO:0021788chemoattraction of branchiomotor neuron axon in neural tube

GO:0021789branchiomotor neuron axon guidance in branchial arch mesenchyme

GO:0021785branchiomotor neuron axon guidance

GO:0021786branchiomotor neuron axon guidance in neural tube

GO:0021787chemorepulsion of branchiomotor neuron axon in neural tube

GO:0021791chemoattraction of branchiomotor neuron axon in branchial arch mesenchyme

GO:0021792chemoattraction of branchiomotor axon

GO:0021793chemorepulsion of branchiomotor axon

GO:0021790chemorepulsion of branchiomotor neuron axon in branchial arch mesenchyme

GO:0021623oculomotor nerve formation

GO:0021624oculomotor nerve structural organization

GO:0021625oculomotor nerve maturation

GO:0021620hypoglossal nerve formation

GO:0021621hypoglossal nerve structural organization

GO:0021622oculomotor nerve morphogenesis

GO:0021616glossopharyngeal nerve formation

GO:0021617glossopharyngeal nerve structural organization

GO:0021618hypoglossal nerve morphogenesis

GO:0021619hypoglossal nerve maturation

GO:0021635trigeminal nerve maturation

GO:0021636trigeminal nerve morphogenesis

GO:0021637trigeminal nerve structural organization

GO:0021641trochlear nerve structural organization

GO:0021642trochlear nerve formation

GO:0021640trochlear nerve maturation

GO:0021638trigeminal nerve formation

GO:0021639trochlear nerve morphogenesis

GO:2001049regulation of tendon cell differentiation

GO:2001050negative regulation of tendon cell differentiation

GO:2001051positive regulation of tendon cell differentiation

GO:1903951positive regulation of AV node cell action potential

GO:1903950negative regulation of AV node cell action potential

GO:0086027AV node cell to bundle of His cell signaling

GO:0086028bundle of His cell to Purkinje myocyte signaling

GO:0086015SA node cell action potential

GO:0086016AV node cell action potential

GO:0086019cell-cell signaling involved in cardiac conduction

GO:0086063voltage-gated sodium channel activity involved in SA node cell action potential

GO:0086060voltage-gated sodium channel activity involved in AV node cell action potential

GO:0086061voltage-gated sodium channel activity involved in bundle of His cell action potential

GO:0086069bundle of His cell to Purkinje myocyte communication

GO:0086052membrane repolarization during SA node cell action potential

GO:0086050membrane repolarization during bundle of His cell action potential

GO:0086056voltage-gated calcium channel activity involved in AV node cell action potential

GO:0086053AV node cell to bundle of His cell communication by electrical coupling

GO:0086054bundle of His cell to Purkinje myocyte communication by electrical coupling

GO:0086059voltage-gated calcium channel activity involved SA node cell action potential

GO:0086057voltage-gated calcium channel activity involved in bundle of His cell action potential

GO:0086041voltage-gated potassium channel activity involved in SA node cell action potential depolarization

GO:0086045membrane depolarization during AV node cell action potential

GO:0086043bundle of His cell action potential

GO:0086048membrane depolarization during bundle of His cell action potential

GO:0086049membrane repolarization during AV node cell action potential

GO:0086046membrane depolarization during SA node cell action potential

GO:0086086voltage-gated potassium channel activity involved in AV node cell action potential repolarization

GO:0048483autonomic nervous system development

GO:0050885neuromuscular process controlling balance

GO:0050882voluntary musculoskeletal movement

GO:0050881musculoskeletal movement

GO:0050804modulation of chemical synaptic transmission

GO:0050803regulation of synapse structure or activity

GO:0050806positive regulation of synaptic transmission

GO:0050805negative regulation of synaptic transmission

GO:0050808synapse organization

GO:0050807regulation of synapse organization

GO:0051450myoblast proliferation

GO:0051451myoblast migration

GO:0051625epinephrine uptake

GO:0051373FATZ binding

GO:0051379epinephrine binding

GO:0051932synaptic transmission, GABAergic

GO:0007498mesoderm development

GO:0007422peripheral nervous system development

GO:0007504larval fat body development

GO:0007518myoblast fate determination

GO:0007520myoblast fusion

GO:0007529establishment of synaptic specificity at neuromuscular junction

GO:0033292T-tubule organization

GO:0008031eclosion hormone activity

GO:0005308creatine transmembrane transporter activity

GO:1900383regulation of synaptic plasticity by receptor localization to synapse

GO:0030314junctional membrane complex

GO:0030315T-tubule

GO:0030239myofibril assembly

GO:0005523tropomyosin binding

GO:0005861troponin complex

GO:0005884actin filament

GO:0044616venom-mediated paralysis

GO:0030017sarcomere

GO:0030016myofibril

GO:0030018Z disc

GO:0042418epinephrine biosynthetic process

GO:0042419epinephrine catabolic process

GO:0042414epinephrine metabolic process

GO:0042310vasoconstriction

GO:0042311vasodilation

GO:0042383sarcolemma

GO:0042396phosphagen biosynthetic process

GO:0042397phosphagen catabolic process

GO:0044291cell-cell contact zone

GO:0019230proprioception

GO:0044221host cell synapse

GO:0044231host cell presynaptic membrane

GO:0017166vinculin binding

GO:0003014renal system process

GO:0003057regulation of the force of heart contraction by chemical signal

GO:0003058hormonal regulation of the force of heart contraction

GO:0003059positive regulation of the force of heart contraction by epinephrine

GO:0003060negative regulation of the force of heart contraction by acetylcholine

GO:0003061positive regulation of the force of heart contraction by norepinephrine

GO:0003063negative regulation of heart rate by acetylcholine

GO:0003064regulation of heart rate by hormone

GO:0003065positive regulation of heart rate by epinephrine

GO:0003066positive regulation of heart rate by norepinephrine

GO:0003222ventricular trabecula myocardium morphogenesis

GO:0003223ventricular compact myocardium morphogenesis

GO:0003248heart capillary growth

GO:0003262endocardial progenitor cell migration to the midline involved in heart field formation

GO:0003108negative regulation of the force of heart contraction by chemical signal

GO:0003109positive regulation of the force of heart contraction by circulating norepinephrine

GO:0003110positive regulation of the force of heart contraction by neuronal norepinephrine

GO:0003111positive regulation of heart rate by circulating epinephrine

GO:0003112positive regulation of heart rate by neuronal epinephrine

GO:0003113positive regulation of heart rate by neuronal norepinephrine

GO:0003114positive regulation of heart rate by circulating norepinephrine

GO:0042734presynaptic membrane

GO:0003163sinoatrial node development

GO:0003164His-Purkinje system development

GO:0003165Purkinje myocyte development

GO:0003168Purkinje myocyte differentiation

GO:0003087positive regulation of the force of heart contraction by neuronal epinephrine

GO:0003088positive regulation of the force of heart contraction by circulating epinephrine

GO:0003089positive regulation of the force of heart contraction by circulating epinephrine-norepinephrine

GO:0003090positive regulation of the force of heart contraction by neuronal

epinephrine-norepinephrine

GO:0003099positive regulation of the force of heart contraction by chemical signal

GO:0002353plasma kallikrein-kinin cascade

GO:0097086amniotic stem cell differentiation

GO:0097090presynaptic membrane organization

GO:0097512cardiac myofibril

GO:0097476spinal cord motor neuron migration

GO:0097475motor neuron migration

GO:0097195pilomotor reflex

GO:0097120receptor localization to synapse

GO:0097105presynaptic membrane assembly

GO:0097049motor neuron apoptotic process

GO:0097060synaptic membrane

GO:0098521inhibitory neuromuscular junction

GO:0098520excitatory neuromuscular junction

GO:0098523neuromuscular junction of myotube

GO:0098525excitatory neuromuscular junction of somatic myotube

GO:0098526inhibitory neuromuscular junction of somatic myotube

GO:0098736negative regulation of the force of heart contraction

GO:0098735positive regulation of the force of heart contraction

GO:0043034costamere

GO:0043051regulation of nematode pharyngeal pumping

GO:0043050nematode pharyngeal pumping

GO:0070514SRF-myogenin-E12 complex

GO:0098905regulation of bundle of His cell action potential

GO:0098904regulation of AV node cell action potential

GO:0098907regulation of SA node cell action potential

GO:0098975postsynapse of neuromuscular junction

GO:0043134regulation of hindgut contraction

GO:0043133hindgut contraction

GO:0045445myoblast differentiation

GO:0045214sarcomere organization

GO:0045202synapse

GO:0045159myosin II binding

GO:0002254kinin cascade

GO:0002255tissue kallikrein-kinin cascade

GO:0016013syntrophin complex

GO:0016010dystrophin-associated glycoprotein complex

GO:0016011dystroglycan complex

GO:0055004atrial cardiac myofibril assembly

GO:0055005ventricular cardiac myofibril assembly

GO:0055003cardiac myofibril assembly

GO:0002162dystroglycan binding

GO:0002086diaphragm contraction

GO:0016084myostimulatory hormone activity

GO:0016085myoinhibitory hormone activity

GO:0016528sarcoplasm

GO:0016529sarcoplasmic reticulum

GO:0016460myosin II complex

GO:0016461unconventional myosin complex

GO:00046451,4-alpha-oligoglucan phosphorylase activity

GO:0004687myosin light chain kinase activity

GO:0045663positive regulation of myoblast differentiation

GO:0045661regulation of myoblast differentiation

GO:0045662negative regulation of myoblast differentiation

GO:0006599phosphagen metabolic process

GO:0031594neuromuscular junction

GO:0031433telethonin binding

GO:0031432titin binding

GO:0031430M band

GO:0070888E-box binding

GO:0031673H zone

GO:0006603phosphocreatine metabolic process

GO:0006600creatine metabolic process

GO:0006601creatine biosynthetic process

GO:0006604phosphoarginine metabolic process

GO:0044499venom-mediated vasoconstriction

GO:0007268chemical synaptic transmission

GO:0007014actin ubiquitination

GO:0044522venom-mediated myocyte killing in another organism

GO:0044523venom-mediated disruption of extracellular matrix in another organism

GO:0044551venom-mediated vasodilation

GO:0071119alpha7-beta1 integrin-nicotinamide riboside kinase complex

GO:0044760symbiont-mediated perturbation of host cholinergic synaptic transmission

GO:0044761symbiont-mediated suppression of host cholinergic synaptic transmission

GO:0044759symbiont-mediated suppression of host synaptic transmission

GO:0044758symbiont-mediated perturbation of host synaptic transmission

GO:0007271synaptic transmission, cholinergic

GO:0007274neuromuscular synaptic transmission

GO:1990629phospholamban complex

GO:1990733titin-telethonin complex

GO:1990454L-type voltage-gated calcium channel complex

GO:1990425ryanodine receptor complex

GO:1990584cardiac Troponin complex

GO:1990566I(KACh) inward rectifier potassium channel complex

GO:1990320collagen type XXI trimer

GO:0001997positive regulation of the force of heart contraction by epinephrine-norepinephrine

GO:0001986negative regulation of the force of heart contraction involved in baroreceptor response to increased systemic arterial blood pressure

GO:0001963synaptic transmission, dopaminergic

GO:0001821histamine secretion

GO:1990014orthogonal array

GO:0014715myoblast fate commitment in trunk

GO:0014714myoblast fate commitment in head

GO:0000146microfilament motor activity

GO:0014866skeletal myofibril assembly

GO:0014872myoblast division

GO:0014886transition between slow and fast fiber

GO:0014883transition between fast and slow fiber

GO:0014882regulation of myofibril number

GO:0014881regulation of myofibril size
