# Supplementary material for: Establishment and Characterization of an Immortalized Porcine Satellite Cell Line from China Junmu No.1 Pigs
Source: Vet Sci. 2026 Jun 4;13(6):556. doi: 10.3390/vetsci13060556 (PMC13308346; doi:10.3390/vetsci13060556)
Supplement: Supplementary file 1 [file vetsci-13-00556-s001.zip › Supplementary File S4-primer list.pdf]

Supplement File S4 : qPCR primer sequences for each target gene

gene name	sequence
PAX3	F 5′- GCAGCACCGTTCACAGACCT-3′
	R 5′-CGGGGTTCATGGGGTTGGAG-3′
MYOD1	F 5′-AACTGTTCCGACGGCATGAT-3′
	R 5′-AGATGCTCTCCACGATGCTG-3′
MYOG	F 5′-CTCCTGCAGTCCAGAATGGG-3′
	R 5′- GGGCATGGTTTCATCTGGGA-3′
MYH3(MYHC)	F 5′- CTCCTCACGCTTTGGTAACT-3′
	R 5′- GTGATTTCTTCTGTCACCTTAGGT-3′
GAPDH	F 5′-CAGCGGTACGAGATCCTGAC-3′
	R 5′- TTGGGATCCAATTCCAGGGC-3′
